# Epigenetic drug screening for trophoblast syncytialization reveals a novel role for MLL1 in regulating fetoplacental growth

**DOI:** 10.1186/s12916-024-03264-8

**Published:** 2024-02-05

**Authors:** Jiayi Wu, Chuanmei Qin, Fuju Tian, Xueqing Liu, Jianing Hu, Fan Wu, Cailian Chen, Yi Lin

**Affiliations:** 1 The International Peace Maternity and Child Health Hospital, School of Medicine, Shanghai Jiao Tong University, Shanghai, China; 2 Shanghai Key Laboratory of Embryo Original Diseases, Shanghai, China; 3 Institute of Birth Defects and Rare Diseases, School of Medicine, Shanghai Jiao Tong University, Shanghai, China; 4 Shanghai First Maternity and Infant Hospital, Tongji University School of Medicine, Shanghai, China; 5 Department of Automation, Shanghai Jiao Tong University, Key Laboratory of System Control and Information Processing, Ministry of Education of China, Shanghai, China; 6 Shanghai Jiao Tong University Affiliated Sixth People’s Hospital, Shanghai, China

**Keywords:** Trophoblast syncytialization, Placenta, MLL1, H3K4me3, TEAD4

## Abstract

**Background:**

Abnormal placental development is a significant factor contributing to perinatal morbidity and mortality, affecting approximately 5–7% of pregnant women. Trophoblast syncytialization plays a pivotal role in the establishment and maturation of the placenta, and its dysregulation is closely associated with several pregnancy-related disorders, including preeclampsia and intrauterine growth restriction. However, the underlying mechanisms and genetic determinants of syncytialization are largely unknown.

**Methods:**

We conducted a systematic drug screen using an epigenetic compound library to systematically investigate the epigenetic mechanism essential for syncytialization, and identified mixed lineage leukemia 1 (MLL1), a histone 3 lysine 4 methyltransferase, as a crucial regulator of trophoblast syncytialization. BeWo cells were utilized to investigate the role of MLL1 during trophoblast syncytialization. RNA sequencing and CUT&Tag were further performed to search for potential target genes and the molecular pathways involved. Human placenta tissue was used to investigate the role of MLL1 in TEA domain transcription factor 4 (TEAD4) expression and the upstream signaling during syncytialization. A mouse model was used to examine whether inhibition of MLL1-mediated H3K4me3 regulated placental *TEAD4* expression and fetoplacental growth.

**Results:**

Genetic knockdown of *MLL1* or pharmacological inhibition of the MLL1 methyltransferase complex (by MI-3454) markedly enhanced syncytialization, while overexpression of *MLL1* inhibited forskolin (FSK)-induced syncytiotrophoblast formation. In human placental villous tissue, MLL1 was predominantly localized in the nuclei of cytotrophoblasts. Moreover, a notable upregulation in MLL1 expression was observed in the villus tissue of patients with preeclampsia compared with that in the control group. Based on RNA sequencing and CUT&Tag analyses, depletion of MLL1 inhibited the Hippo signaling pathway by suppressing TEAD4 expression by modulating H3K4me3 levels on the *TEAD4* promoter region. *TEAD4* overexpression significantly reversed the FSK-induced or *MLL1* silencing-mediated trophoblast syncytialization. Additionally, decreased hypoxia-inducible factor 1A (HIF1A) enrichment at the *MLL1* promoter was observed during syncytialization. Under hypoxic conditions, HIF1A could bind to and upregulate MLL1, leading to the activation of the MLL1/TEAD4 axis. In vivo studies demonstrated that the administration of MI-3454 significantly enhanced fetal vessel development and increased the thickness of the syncytial layer, thereby supporting fetoplacental growth.

**Conclusions:**

These results revealed a novel epigenetic mechanism underlying the progression of syncytialization with MLL1, and suggest potential avenues for identifying new therapeutic targets for pregnancy-related disorders.

**Supplementary Information:**

The online version contains supplementary material available at 10.1186/s12916-024-03264-8.

## Background

Abnormal placental growth is a common cause of perinatal morbidity and mortality, affecting approximately 5–7% of pregnant women [1, 2]. It is also considered as a potential reason for diverse pregnancy complications, such as intrauterine growth restriction (IUGR), preeclampsia (PE), and miscarriage [3, 4]. More seriously, the resultant heath condition might increase the risk of developing disorders in later life, including hypertension and type 2 diabetes [5–7]. Trophoblast syncytialization is a critical process in placental formation, defined as the fusion of mononuclear cytotrophoblasts (CTBs) into multinucleated syncytiotrophoblasts (STBs). Continuous STBs comprise the syncytial layer, lining the outmost surface of the placenta and controlling the exchange of gases, nutrients, and waste products between maternal and fetal blood. Indeed, insufficient trophoblast syncytialization can severely impair placental function and is a feature of PE and IUGR [8–10]. However, we still lack suitable biomarkers for abnormal syncytialization and placental formation; therefore, a better understanding of the underlying mechanisms is necessary.

Epigenetic mechanisms can regulate gene expression through DNA methylation, histone modifications, and non-coding RNA, and are involved in a wide range of cellular processes. Moreover, epigenetic modifications have been shown to play critical roles in modulating the expression of developmental genes during early embryonic development, decidualization, and embryo implantation in mammals [11–13]. The impressive clinical benefits of drugs targeting specific epigenetic mechanisms has led to a number of epigenetic-related inhibitors or activators entering clinical use or being at various stages of development. Thus, identifying epigenetic targets that are vital for trophoblast syncytialization and placental growth might have translational potential for clinical application. However, apart from some independent studies [14, 15], little is known about the role of epigenetic mechanisms in syncytialization. Thus, there is a compelling need for further research in this area.

Mixed lineage leukemia 1 (MLL1) is one of a family of six histone methyltransferases (HMTs), responsible for catalyzing the trimethylation of H3K4 via a conserved SET [**S**u(var)3–9, **E**nhancer-of-zeste and **T**rithorax] domain. H3K4me3 modification is a well-recognized transcription activating mark, which can loosen chromatin compaction and make the promoter of the target gene more accessible to transcription factors. Disruption of MLL upregulates the expression of *HOXA9* (encoding homeobox A9) and *MEIS1* (encoding Meis homeobox 1), promoting proliferation and inhibiting hematopoietic differentiation. This can ultimately lead to the development of acute leukemia [16–18]. In particular, MLL1, via its methyltransferase activity, plays vital roles in embryonic stem cell development, hematopoiesis, and neurogenesis [19–21]. Considering that fetoplacental development is a precisely regulated process, how MLL1 and its target genes participate in trophoblast syncytialization and placental growth remains unknown.

In the current study, we demonstrated the physiological significance of MLL1 in suppressing STB differentiation using human placental villous tissue, human trophoblast cell lines, and an in vivo mouse model. Our findings suggested the possibility of employing MLL1 inhibitors to promote syncytialization and placental development, which could further reduce the incidence of syncytialization dysregulation-related diseases.

## Methods

### Study participants and samples

Three groups of human placental villus tissue were collected at the International Peace Maternity and Child Health Hospital (IPMCH) of Shanghai Jiao Tong University School of Medicine, with Group 1 being obtained from uncomplicated pregnancies in the first trimester, Group 2 being obtained from uncomplicated pregnancies in the third trimester, and Group 3 being obtained from pregnancies with preeclampsia in the third trimester. First-trimester samples were obtained from women who underwent elective abortion procedures. These patients had a history of at least one previous uncomplicated full-term pregnancy and opted for abortion because of unintended pregnancies. Based on the practice bulletins of The American College of Obstetricians and Gynecologists, PE was diagnosed as diastolic blood pressure ≥ 90 mmHg or systolic blood pressure ≥ 140 mmHg twice after 20 weeks of gestation, with proteinuria or the following conditions: thrombocytopenia, pulmonary edema, impaired liver function, renal dysfunction, or cerebral or visual disturbances. Women with previously diagnosed hypertension, renal diseases, cardiovascular disease, or other pregnancy complications (e.g., gestational thyroid disorders, diabetes mellitus, and intrahepatic cholestasis of pregnancy) were excluded. All the participants provided written informed consent. The study protocol was approved by the Institute Medical Ethics Committee of IPMCH (reference number GKLW2021-17) and was carried out according to the Declaration of Helsinki. In addition, the determination of the sample size for human placental samples in our study was guided by the sample sizes reported in prior relevant literature [22, 23].

### Cell culture

BeWo cells, a human trophoblast cell line exhibiting cytotrophoblastic characteristics [24], were purchased from the National BioMedical Cell-Line Resource (Beijing, China; 4201HUM-CCTCC00072). BeWo cells were cultured in Dulbecco’s modified Eagle’s medium (DMEM)-F12 medium containing 15% fetal bovine serum (Gibco, Grand Island, NY, USA) at 37 °C under a 5% CO_2_ atmosphere. Hypoxic conditions were initiated by culturing 1 × 10^5^ cells per well in a six-well plate of BeWo cells in a hypoxic incubator (with 1% O_2_, 5% CO_2_, at 37 °C) at the designated time points.

JEG3 cells were purchased from the National BioMedical Cell-Line Resource (Beijing, China; 4201HUM-CCTCC00320). JEG3 cells were cultured in DMEM medium containing 10% fetal bovine serum at 37 °C under a 5% CO_2_ atmosphere.

### Drug screening

An epigenetic compound library was purchased from MedChemExpress screening libraries (Monmouth Junction, NJ, USA; Catalog No.: HYCPK16202-16203) which targeted, for example, histone deacetylases, histone demethylases, histone acetyltransferases, DNA methyltransferases, and epigenetic reader domains. BeWo cells were treated with relatively low concentrations of each drug according to the supplier's instructions for 24 h (Additional file 1: Table S1), and cells treated with dimethyl sulfoxide (DMSO) were used as the control group. After the extraction of total RNA, relative *HCG* (encoding human chorionic gonadotropin) and *ERVW-1* (encoding endogenous retrovirus group W member 1, envelope, also known as Syncytin 1) mRNA levels were detected to measure the degree of syncytialization using qRT-PCR. The protein–protein interaction (PPI) analysis was performed using STRING version 9.1[25]. In the STRING analysis, edges denoted protein–protein associations that were intended to be both specific and meaningful. These associations were derived from curated databases or experimentally determined. Additionally, methodologies such as gene neighborhood, gene fusions, gene co-occurrence, text mining, co-expression analysis, and consideration of protein homology, were employed to further enhance the understanding of these protein–protein associations.

### RNA extraction and qRT-PCR

Total RNA of tissues or cells was extracted using the TRIzol reagent (Life Technologies, Carlsbad, CA, USA) based on the manufacturer’s instructions. Agarose gel electrophoresis was employed to assess the integrity of the RNA. A 28S band with an intensity approximately twice that of the 18S band was used for a preliminary assessment of relatively intact total RNA. The quality and concentration of the RNA were detected using a NanoDrop 2000c spectrophotometer (Thermo Fisher Scientific, Waltham, MA, USA), and 1 μg of RNA was reverse transcribed into cDNA using an Evo M-MLV RT Mix Kit with gDNA Clean (Accurate Biology, Hunan, China). A SYBR Green Kit (Accurate Biology) was used for the qPCR step of the qRT-PCR protocol. Relative mRNA expression levels were analyzed using the 2-ΔCT method [26] and standardized against the expression of *ACTB* (encoding beta actin). All primer sequences used in this study are listed in Additional file 2: Table S2 and S3.

### Western blotting

Tissues and cells were lysed using Radioimmunoprecipitation assay (RIPA) buffer containing a protease inhibitor cocktail (Sigma Aldrich, St. Louis, MO, USA), and the protein concentration of each sample was determined using a bicinchoninic acid (BCA) Protein Assay Kit (Pierce Biotechnology, Rockford, IL, USA). Proteins were resolved using sodium dodecyl sulfate–polyacrylamide gel electrophoresis (SDS-PAGE) and electro-transferred to polyvinylidene fluoride (PVDF) membranes. After blocking, the membranes were incubated with primary antibodies against MLL1 (1:1000 dilution, Cell Signaling Technology, Danvers, MA, USA; 14,197), Menin (1:1000 dilution, Abcam, Cambridge, MA, USA; ab92443), H3K4me3 (1:1000 dilution, Cell Signaling Technology; 9751), glial cells missing transcription factor 1 (GCM1) (1:1000 dilution, Proteintech, Rosemont, IL, USA; 21,724–1-AP), HCG (1:1000 dilution, Proteintech, 11,615–1-AP), TEA domain transcription factor 4 (TEAD4) (1:1000 dilution, Proteintech, 12,418–1-AP), glyceraldehyde-3-phosphate dehydrogenase (GAPDH) (1:5000 dilution, Proteintech, 10,494–1-AP), Actin (1:5000 dilution, Proteintech, 20,536–1-AP), and histone 3 (H3) (1:5000 dilution, Proteintech, 17,168–1-AP). The membranes were then incubated with the appropriate horseradish peroxidase (HRP)-conjugated secondary antibody and visualized using an HRP chemiluminescent kit (Millipore, Billerica, MA, USA). The density of all immunoreactive protein bands was evaluated using Image J software (NIH, Bethesda, MD, USA).

### Gene knockdown and overexpression

BeWo cells were seeded onto 6-well plates at 100,000 cells per well and transfected using the JetPRIME reagent (Polyplus-transfection SA, Strasbourg, France) with small interfering RNAs (siRNAs) targeting specific genes or a nonspecific scrambled siRNA as a negative control (si-NC) (Gene Pharma Co., Shanghai, China). The sequences of the siRNAs were as follows: *MLL1*, GGAUCAGAGUGGACUUUAATT (sense) and UUAAAGUCCACUCUGAUCCTT (antisense). To overexpress *MLL1*, the coding sequence was ligated into a plasmid (Addgene plasmid, Addgene, Watertown, MA, USA; # 20,873) and then transiently transfected into BeWo cells using the JetPRIME reagent. A plasmid including the coding region of human *TEAD4* was designed and purchased from VectorBuilder (VectorBuilder Inc., Guangdong, China). At 48 h after transfection, the cells were collected for RNA or protein extraction and analyzed using qRT-PCR or western blotting.

### Histological analysis

Tissues were fixed using 4% paraformaldehyde, embedded in paraffin, cut into 5 μm thick paraffin sections, and then mounted on glass slides. For human placental samples, immunohistochemical staining was performed using the EXPOSE Mouse and Rabbit-specific HRP/DAB Detection IHC kit (Abcam), as described previously [23]. Primary antibodies against MLL1 (1:50 dilution, Abcam; ab32400), HCG (1:300 dilution, Proteintech; 11,615–1-AP), TEAD4 (1:200 dilution, Abcam; ab97460), and hypoxia-inducible factor 1A (HIF1A) (1:100 dilution, Abcam; ab51608) were applied for incubation, followed by incubation with the corresponding secondary antibodies. Phosphate-buffered saline (PBS) instead of the primary antibody was used as the negative control.

For the mouse placenta samples, hematoxylin and eosin (H&E) staining was performed following standard procedures. Anti-platelet and endothelial cell adhesion molecule 1 (PECAM1, also known as CD31) antibodies (1:2000 dilution, Abcam; ab182981) were used to label fetal endothelial cells via immunohistochemical staining. Monocarboxylate transporter 1 (MCT1) is a specific marker of the apical plasma membrane of STB-I, while MCT4 is a marker of basal plasma membrane of STB-II. For immunofluorescence staining, paraffin sections were incubated with anti-MCT1 antibodies (1:100 dilution, Proteintech; 20,139–1-AP), which stains fused Syn I, the first layer of STBs facing the maternal blood sinuses, and anti-MCT4 (1:50 dilution, Santa Cruz Biotechnology, Santa Cruz, CA, USA; sc-376140), which stains fused Syn II, the second layer of STBs facing the fetal blood vessels, followed by Alexa Fluor 488-donkey anti-chicken antibodies (1:300 dilution, Life technologies, A21206) and Alexa Fluor 594-donkey anti-mouse antibodies (1:300 dilution, Life technologies, A21203). The cell nuclei were stained using 4′,6-diamidino-2-phenylindole (DAPI).

### Animal study

C57BL/6 mice were purchased from Shanghai Laboratory Animal Center, Chinese Academy of Science. Female mice aged 8 to 10 weeks were mated with fertile male mice (8 weeks old), and the day when a vaginal plug was detected was recorded as embryonic day 0.5 (E0.5). A total of 24 pregnant mice were further divided to two groups, with 12 mice in the control group and 12 mice in the MI-3454 (MedChemExpress; Catalog No.: HY-136360) group. The MI-3454 group were injected with MI-3454 at 15 mg/kg/d from E12.5 to E17.5, intravenously, while the control group were injected with an equal amount of solvent (physiological saline containing 2% DMSO, 20% [2-Hydroxypropyl)-β-cyclodextrin (MedChemExpress; Catalog No.: HY-101103), and 5% Cremophore (MedChemExpress; Catalog No.: HY-Y1890)]. The mice were sacrificed by cervical dislocation at E18.5, and the weights of the fetuses and placentas were recorded. After weighing, the placentas were collected for further analysis. The animal study was approved by the Ethics Committee of IPMCH and performed in accordance with the ARRIVE guidelines.

### Transmission electron microscopy

Freshly collected mouse placental tissues from the umbilical cord insertion were used for transmission electron microscopy (TEM). Three placentas from each group (treated with MI-3454 or vehicle) were included in the study. The collected tissues were rapidly immersed in electron microscopy fixation solution (Biossci, Wuhan, China; BP0130). Following standard dehydration, the tissues were embedded in epoxy resin and prepared as ultrathin sections. The sections were then stained with uranyl acetate and lead citrate before being observed using an HT7700 transmission electron microscope (Hitachi, Tokyo, Japan).

### CUT&Tag experiments and Chromatin Immunoprecipitation (ChIP)

The library preparation for CUT&Tag was performed using a Hyperactive® Universal CUT&Tag Assay Kit for Illumina (Vazyme, Nanjing, China; TD903) according to the manufacturer’s instructions. In brief, 100,000 BeWo cells were collected, washed twice, and then incubated with concanavalin A-coated magnetic beads for 10 min at room temperature. After collection, the cell-bound beads were incubated overnight at 4 °C with antibodies recognizing MLL1 (1:50 dilution, Abcam; ab272023), H3K4me3 (1:50 dilution, Cell Signaling Technology; 9751), HIF1A (1:50 dilution, Abcam; ab51608) or Rabbit IgG monoclonal antibodies (mAbs) (1:50 dilution, Cell Signaling Technology; 66,362). Following removal of the supernatant using a magnet stand, the cells were further incubated with secondary antibodies (1:50 dilution, Abcam; ab6702) for 60 min at room temperature. The Hyperactive pA/G-Transposon incubation, tagmentation, and amplification steps were performed as reported previously [27]. VAHTS DNA Clean Beads (Vazyme, N411) were applied to purify the DNA libraries. The CUT&Tag sequencing was performed and analyzed by Genewiz (Jiangsu, China).

A SimpleChIP® Enzymatic Chromatin IP Kit (Cell Signaling Technology, 9003) was used for the ChIP assays. In short, BeWo cells were transfected with siMLL1 or treated with Forskolin (FSK) (25 μM) (Sigma, F6886) for 48 h and fixed using formaldehyde. After stopping the fixation with glycine (0.125 M), chromatin was digested into fragments with an average length of 500 bp and incubated with the primary antibodies at 4 °C overnight. The immunocomplexes were then captured using Protein G magnetic beads. After reversal of the cross-links, purified DNA was subjected to standard PCR and qPCR analysis. All primers used in the PCR experiments are listed in Additional file 2: Table S2.

### RNA sequencing (RNA-Seq analysis)

Three groups of BeWo cells were used for RNA-Seq analysis, comprising cells transfected with siMLL1, cells transfected with siNC, and cells transfected with siNC and treated with FSK (25 μM). Total RNA of the samples was extracted using 1 mL of TRIzol (Invitrogen) and transported to Novogene (Beijing, China) for further sequencing and analysis. Briefly, the transcriptome library was generated using an NEBNext® Ultra™ RNA library prep kit for Illumina® (NEB, Ipswich, MA, USA) and sequenced on the Illumina Novaseq platform (Illumina, San Diego, CA, USA). After quality control and annotation, differentially expressed genes were determined using DEseq2 R package. Genes with adjusted P values < 0.05 were subjected to Kyoto Encyclopedia of Genes and Genomes (KEGG) and DisGeNET [28] (a platform containing gene-disease and variant-disease associations) enrichment analysis.

### Statistical analysis

Data are presented as mean with the standard deviation. After checking normality and homogeneity of variance, Student’s t-test or a Mann–Whitney test were used for comparison, as appropriate. The correlation analysis was conducted using Spearman’s rank correlation test. All the graphing and statistical analyses were performed using GraphPad Prism 8 (GraphPad Inc., La Jolla, CA, USA). Two-tailed P values < 0.05 were considered significant.

## Results

### Drug screening uncovers MLL1 as a key regulator of syncytialization

**Fig. 1:**
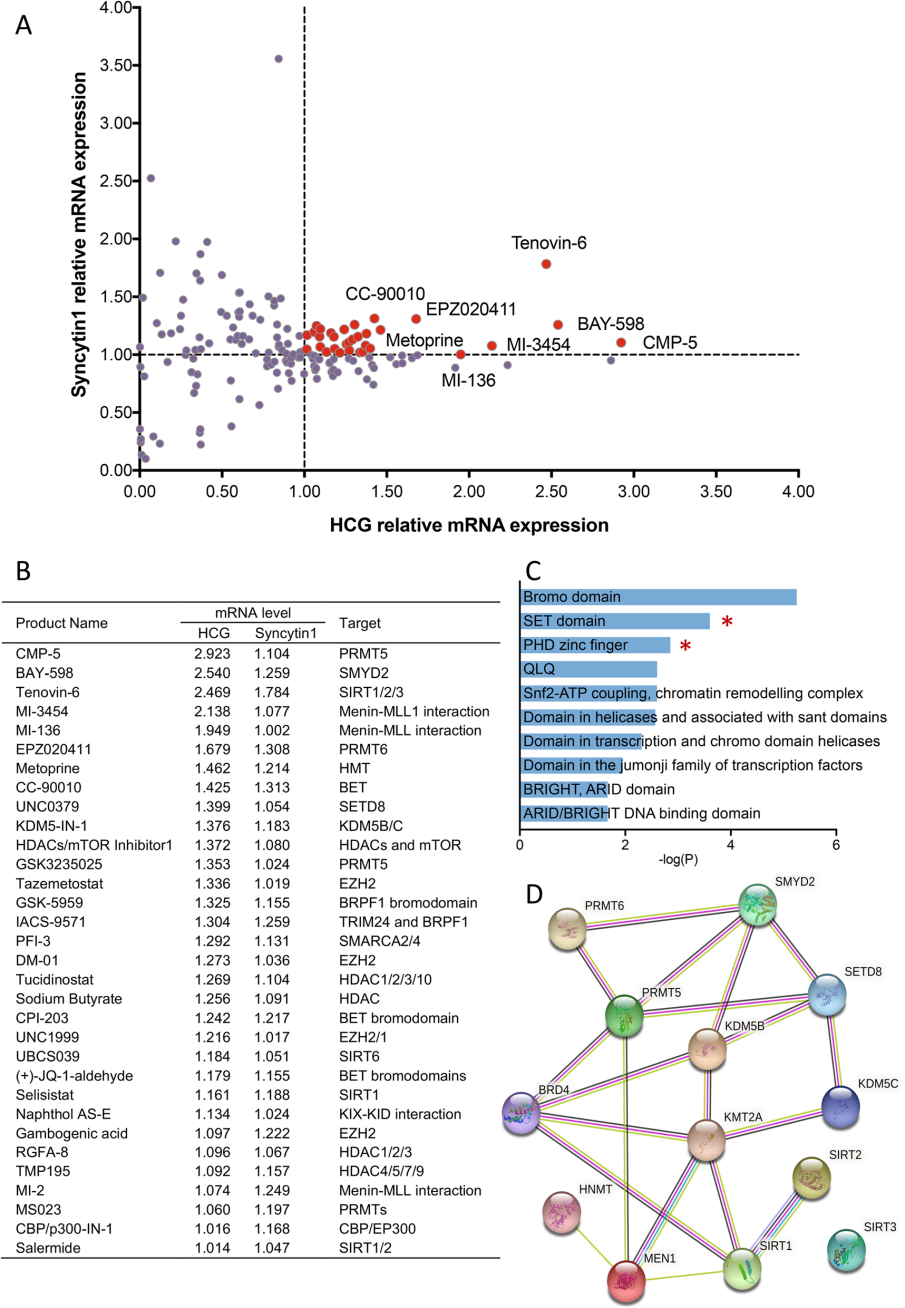
Drug screening identified 32 epigenetic compounds that promote trophoblast syncytialization. **A** Scatter plot displaying the effects of epigenetic drugs on the syncytialization process of BeWo cells. The y-axis and x-axis show the relative mRNA levels of two STB markers after administration of each drug normalized against DMSO control. Each dot represents an individual drug. **B** A list of 32 drugs is shown with their relative mRNA levels of two STB markers and targets. **C** An enrichment analysis of the targets of the 32 drugs based on the SMART database. **D** STRING analysis of all the targets of the 32 drugs based on the protein–protein interaction networks, highlighting the essential role of KMT2A (MLL1) with many connecting nodes among the gene targets promoting trophoblast syncytialization. STB, syncytiotrophoblast; DMSO, dimethyl sulfoxide; MLL1, mixed lineage leukemia 1; KMT2A, lysine methyltransferase 2A

To systematically identify the epigenetic mechanism essential for syncytialization, we performed forward drug screens using an epigenetic compound library containing 160 small molecule modulators. Promotion of syncytialization was defined by elevated mRNA expression of HCG and Syncytin 1, two markers of trophoblast syncytialization [29], compared with that in the DMSO group (Fig. 1A). The top 32 drugs that enhanced syncytialization were determined and subjected to further analyses (Fig. 1B). Our analysis found that targets of certain drugs were associated with the degree of histone methylation. For example, PRMT5 methylates histone H2A and H4 arginine 3 in germ cell development [30], while SMYD2 catalyzes histone lysine methylation and drives differentiation of stem cells [31]. Consistently, SMART database enrichment analysis also suggested that a conserved catalytic domain of methyltransferase, the SET domain, might play an important role in syncytialization (Fig. 1C).

To explore whether proteins corresponding to drug targets might interact with each other as part of interacting networks, a STRING analysis was conducted [25] (Fig. 1D). Surprisingly, the result showed that KMT2A (lysine methyltransferase 2A, also known as MLL1) was the most connected “node” with the largest number of interactions with other gene products. MLL1-mediated H3K4me3 is a critical modification for transcriptional activation, which has been implicated in regulating multiple cell differentiations [21, 32, 33], prompting us to further examine and verify the role of MLL1 during syncytialization.

In addition, to further validate our screening results, immunofluorescence experiments were performed using the top 10 drugs that exhibited the most significant enhancement of syncytialization to directly evaluate their impact on cell morphology. The results confirmed that these 10 drugs effectively stimulated the fusion of BeWo cells, consistent with our qRT-PCR findings (Additional file 2: Fig. S1A). Along with FSK treatment, the top 10 drugs effectively enhanced the levels of the STB biomarkers (HCG and Syncytin 1) (Additional file 2: Fig. S1B-C). Specifically, the drug MI-3454 resulted in a 1.35-fold increase in *HCG* and a 1.24-fold increase in *Syncytin 1* mRNA levels.

### The role of MLL1-mediated H3K4me3 in regulating syncytialization

**Fig. 2:**
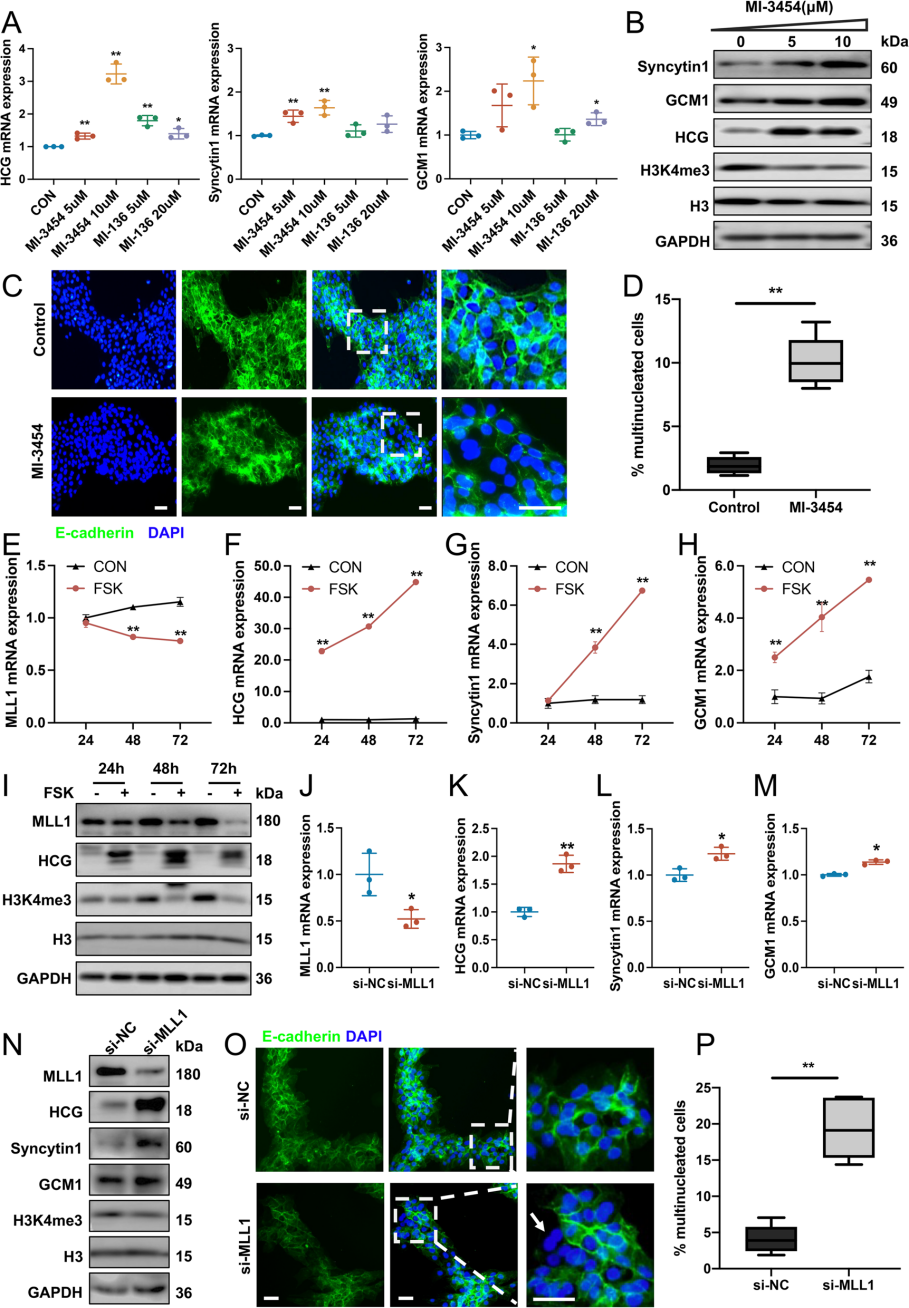
Validation of MLL1 as a negative mediator of trophoblast syncytialization. **A** mRNA levels of STB markers (HCG, Syncytin 1 and GCM1) in BeWo cells treated with MLL1 pathway inhibitors. The values are normalized to that of *ACTIN* and indicated as the mean ± SD (*n* = 3 biologically independent samples). **B** Western blots of Syncytin 1, GCM1, HCG, and H3K4me3 in BeWo cells exposed to 0, 5, and 10 μM MI-3454. **C**, **D** Immunostaining of E-cadherin (green) and DAPI (blue) (**C**) and corresponding quantification of multinucleated cells (**D**) among BeWo cells exposed to MI-3454 or DMSO. Scale bar, 40 μm. **E**–**H** mRNA levels of *MLL1* and STB markers in BeWo cells treated with or without 25 μM FSK for 24, 48, and 72 h. **I** Western blots of MLL1, HCG, and H3K4me3 in BeWo cells treated with or without 25 μM FSK for 24, 48, and 72 h. **J-N** mRNA levels of *MLL1* and STB markers (J-M) and western blots (N) of MLL1, Syncytin 1, GCM1, HCG, and H3K4me3 in BeWo cells transfected with si-MLL1 or si-NC. **O-P** Immunostaining of E-cadherin (green) and DAPI (blue) (**O**) and corresponding quantification of multinucleated cells (**P**) among BeWo cells transfected with si-MLL1 or si-NC. Scale bar, 40 μm. Data are presented as the means ± SD. **P < 0.01, *P < 0.05. CON, control; HCG, human chorionic gonadotropin; GCM1, glial cells missing transcription factor 1; H3k4me3, histone 3 lysine 4 trimethylation; H3 histone 3; GAPDH, glyceraldehyde-3-phosphate dehydrogenase; DAPI, 4′,6-diamidino-2-phenylindole; FSK, forskolin; si-NC, negative control small interfering RNA; si-MLL1, small interfering RNA targeting *MLL1*

BeWo cells were first treated with different concentrations of two Menin-MLL interaction inhibitors (MI-3454 and MI-136), and the expression levels of HCG, Syncytin 1, and GCM1 were examined. The results showed that MI-3454 treatment had a strong promoting effect on trophoblast syncytialization (Fig. 2A). Moreover, MI-3454 treatment significantly increased the protein levels of HCG, Syncytin 1, and GCM1 and decreased the level of trimethylation modification of H3K4 (Fig. 2B and Additional file 2: Fig. S2A-D). Immunofluorescence experiments further verified the effect of MI-3454 on trophoblast morphology by labeling E-cadherin. The ratio of multinucleated cells, defined as cells with more than two nuclei, was significantly higher in the MI-3454 group than in the control group (Fig. 2C and D). The effect of the small molecule inhibitor MI-3454 on MLL1 and Menin expression was examined by western blotting, which showed that MLL1 and Menin levels gradually decreased with increasing doses of MI-3454 (Additional file 2: Fig. S2E-G). This might have been because the small molecule inhibitor triggers protein degradation via the ubiquitin–proteasome pathway [34].

FSK is a protein kinase A agonist that can induce the differentiation of BeWo cells into multinucleated syncytial cells [8]. After treatment of FSK (25 μM) for 24, 48 and 72 h, we found that the mRNA and protein levels of MLL1 and the H3K4me3 modification levels were progressively downregulated, accompanied by increased expression of STB markers (Fig. 2E-I and Additional file 2: Fig. S2H-J). In double fluorescence staining experiments, MLL1 was localized in the nucleus and the MLL1 protein level decreased significantly with the development of syncytialization (Additional file 2: Fig. S2K).

Considering the possibility of off-target effects during the application of inhibitors, the expression of *MLL1* was further silenced using a specific siRNA or overexpressed from a plasmid. The knockdown and overexpression efficiencies were measured using qRT-PCR and western blotting (Fig. 2J and N, Additional file 2: Fig. S2L, Additional file 2: Fig. S3A-C). The results showed that *MLL1* knockdown significantly downregulated the level of H3K4me3 and upregulated the expression of STB markers (HCG, Syncytin 1, and GCM1) (Fig. 2J-N and Additional file 2: Fig. S2M-P). Importantly, an increased proportion of multinucleated cells was observed in cells transfected with siMLL1 (Fig. 2O and P), suggesting that *MLL1* knockdown could promote trophoblast syncytialization. When *MLL1* was overexpressed, FSK-induced HCG, Syncytin 1, and GCM1 expression (Additional file 2: Fig. S3D-L), as well as syncytium formation, were significantly inhibited (Additional file 2: Fig. S3M). Together, these data indicated that the process of syncytialization in human trophoblasts is inversely related to MLL1 expression.

Previous studies have confirmed that the JEG3 cell line also exhibits the ability to undergo syncytialization under FSK induction [35–37]. This phenomenon was subsequently corroborated in our own research (Additional file 2: Fig. S4A-B). Therefore, we employed the JEG3 cell line for validation in in vitro experiments. The results revealed that the MI-3454 treatment (Additional file 2: Fig. S4C-D) or the knockdown of *MLL1* (Additional file 2: Fig. S4E-F) facilitated syncytialization in JEG3 cells, consistent with the observed outcomes in BeWo cells.

### Impaired syncytialization and upregulated MLL1 in placental villous tissue of patients with preeclampsia

**Fig. 3:**
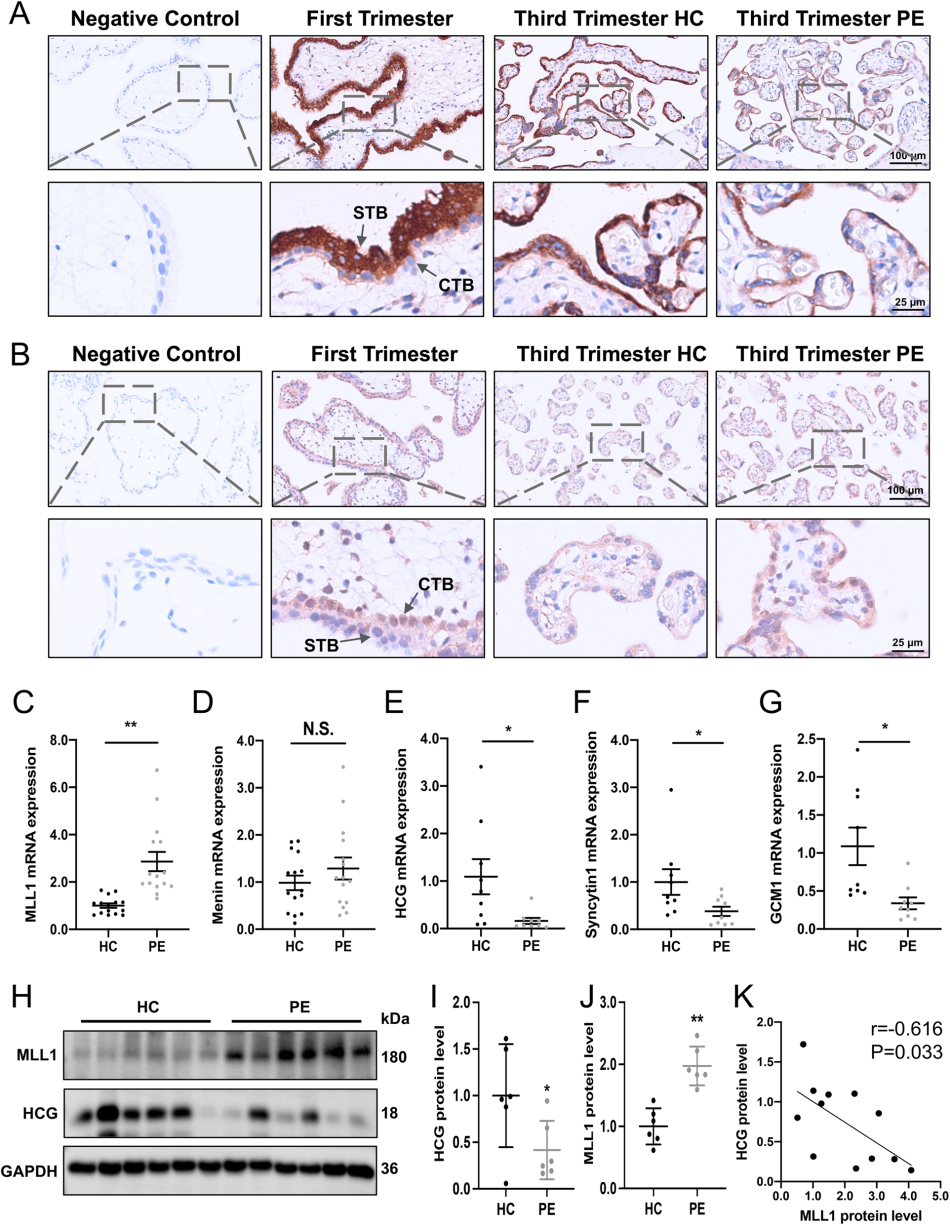
Distribution and levels of MLL1 and STB markers in human placental villi. **A-B** Immunohistochemical analysis of MLL1 (**A**) and HCG (**B**) staining in pregnant women during the first trimester (*n* = 6), third trimester (*n* = 6), and preeclampsia (*n* = 6). **C-G** mRNA levels of *MLL1* (*n* = 15), *MEN1* (*n* = 15), and STB (*n* = 9) markers in women with preeclampsia and healthy controls. **H-J** Western blots (**H**) and corresponding quantification (**I** and **J**) of MLL1 and HCG in the PE (*n* = 6) and control women (*n* = 6). **K** The MLL1 protein level correlated negatively with the protein level of HCG in placental villi. Data are presented as the means ± SD. **P < 0.01, *P < 0.05. HC, healthy control; PE, preeclampsia; CTB, cytotrophoblast; MEN1, menin

Immunohistochemical (IHC) staining was conducted in villous tissue of pregnant women during the first trimester, third trimester, and in those with PE to investigate the localization and expression levels of MLL1 and HCG. HCG localized to the syncytial layer in the first-trimester placenta, and the staining of HCG was significantly less intense in villous tissues from the PE group than in the healthy control (HC) group (Fig. 3A). In contrast to HCG, MLL1 was localized mainly in the nuclei of cytotrophoblasts at early gestation, and increased expression of MLL1 was confirmed in the villus tissue of PE (Fig. 3B). According to the results of qRT-PCR and western blotting, the levels of STB markers were significantly decreased in the human placental villi of the PE group compared with those in the HC group; however, MLL1 expression was significantly increased (Fig. 3C, E-J). The mRNA expression of *MLL1* and *HCG* correlated negatively in villous tissues (r = -0.616 and *P*-value = 0.033) (Fig. 3K). The *MEN1* (encoding Menin) mRNA level was not significantly different between the two groups (Fig. 3D). Collectively, these results suggested that the villous trophoblasts from PE pregnancies are characterized by inadequate syncytialization accompanied by increased expression of MLL1.

### MLL1 downregulation impacts pathways associated with the syncytialization process

**Fig. 4:**
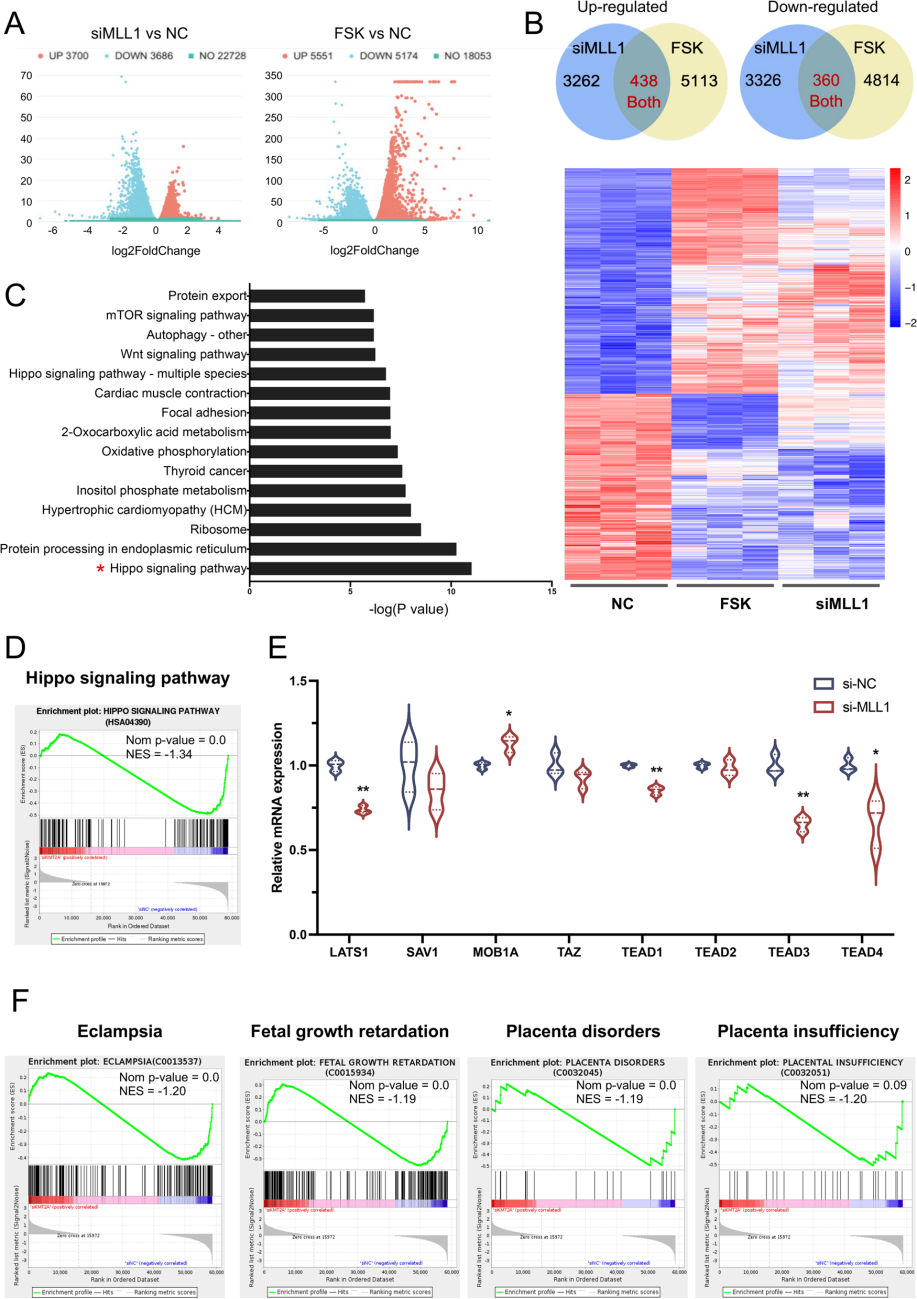
*MLL1* knockdown in BeWo cells leads to downregulation of the Hippo signaling pathway. **A** Volcano plot showing differentially expressed genes after *MLL1* knockdown or FSK treatment. **B** Differentially expressed gene sets with either upregulation or downregulation in both the siMLL and FSK groups. **C** KEGG analyses conducted on 798 genes that were identified as differentially expressed after *MLL1* knockdown and are associated with the syncytialization process. **D** GSEA plot showing the enrichment of Hippo signaling pathway genes in the siMLL group compared with that in the NC group. **E** The relative expression of core regulators in the Hippo signaling pathway. **F** GSEA plot showing the enrichment of diseases related to placental development in the siMLL group compared with those in the NC group. Data are presented as the means ± SD. **P < 0.01. KEGG, Kyoto Encyclopedia of Genes and Genomes; GSEA, gene set enrichment analysis; NC, negative control; NES, normalized enrichment score; LATS1, large tumor suppressor kinase 1; SAV1, salvador family WW domain containing protein 1; MOB1A, MOB kinase activator 1A; TEAD, TEA domain transcription factor

To further explore the potential molecular mechanism by which MLL1 regulates trophoblast syncytialization, three groups of BeWo cells (NC, siMLL1, or FSK-treated) were analyzed for gene expression profiles using RNA-seq. In comparison with the NC group, 7744 differentially expressed genes (DEGs) were identified in cells transfected with siMLL1, and 10,725 DEGs were found in the FSK group (Fig. 4A). Among them, 438 genes were up-regulated and 360 genes were down-regulated in both the siMLL1 and FSK groups, which were regarded as genes aberrantly expressed after *MLL1* knockdown as well as being associated with the syncytialization process (Fig. 4B).

A KEGG pathway analysis was carried out to annotate the functions of these 798 genes. The Hippo signaling pathway, which is considered to play an important role in stem cell function, tissue regeneration, early embryonic development and organ size regulation [38], had the highest score in the enrichment analysis (Fig. 4C). In addition, the DEGs were preferentially enriched in the Wnt and mechanistic target of rapamycin (mTOR) signaling pathways, which are both widely recognized as vital signaling pathways that modulate trophoblast stem cells and cytotrophoblast differentiation [8, 39]. Several biological changes involved in trophoblast syncytialization were also found in the KEGG enrichment analysis, such as cell focal adhesion and autophagy.

Gene set enrichment analysis (GSEA) analysis further clarified that the Hippo signaling pathway was significantly suppressed by *MLL1* knockdown (Fig. 4D). The mRNA levels of key components of the Hippo signaling pathway were confirmed by RT-qPCR. When *MLL1* was silenced, core transcription factor genes including, *TEAD1*, *TEAD3*, and *TEAD4* were downregulated, while *MOB1A* was upregulated, together leading to inhibition of Hippo signaling pathway (Fig. 4E). Based on analysis at DisGeNET, we found that genes associated with eclampsia, fetal growth retardation, placenta disorders, and placenta insufficiency were repressed after *MLL1* knockdown (Fig. 4F), and these findings were consistent with our results in human placental villous tissue.

### MLL1 epigenetically regulates the expression of *TEAD4* in an H3K4me3-dependent manner

**Fig. 5:**
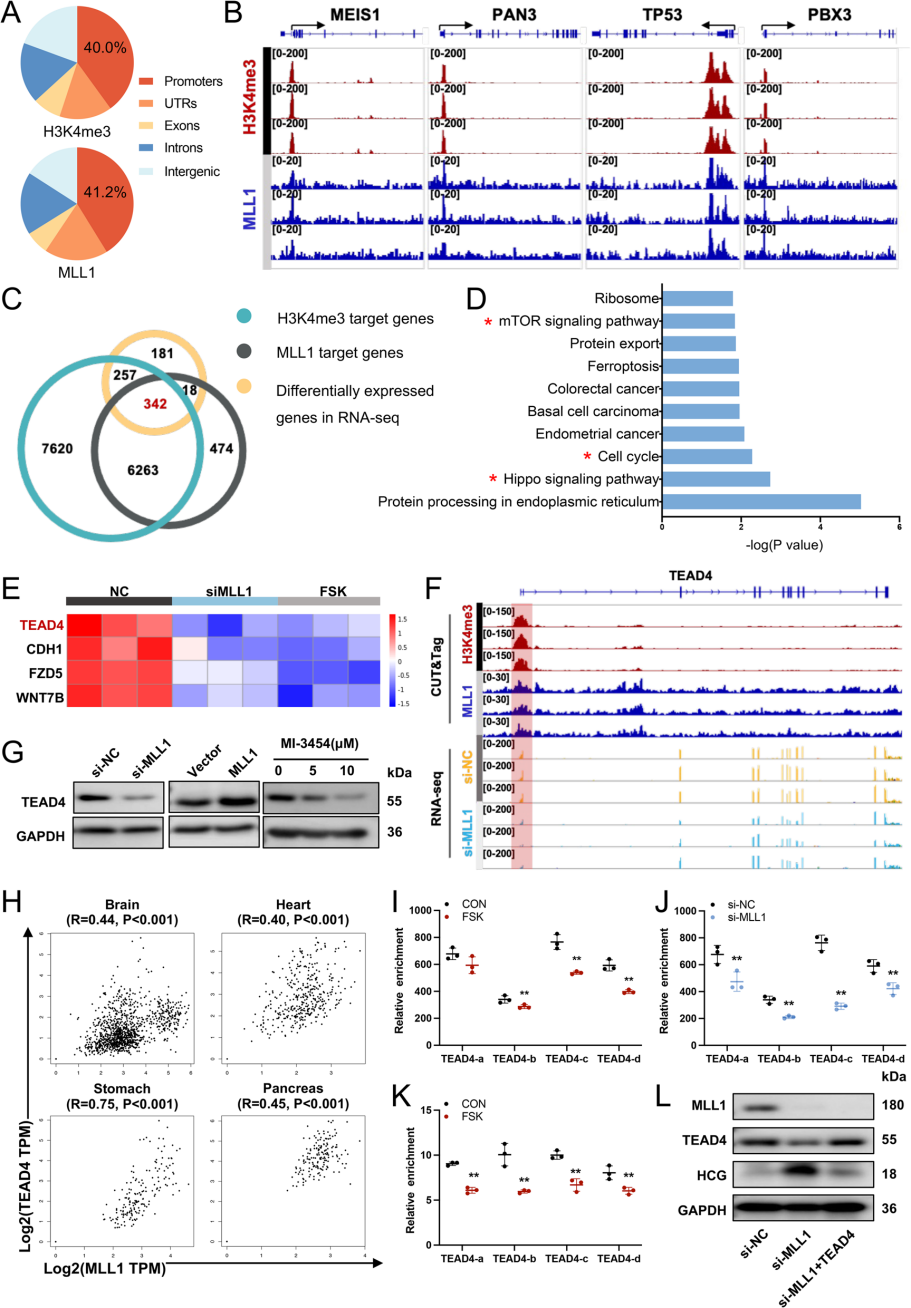
MLL1 regulates trophoblast syncytialization through TEAD4 expression. **A** Genomic distribution of MLL1 and H3K4me3 peaks in BeWo cells. **B** Genome browser view of normalized H3K4me3 and MLL1 CUT&Tag signals for known target genes. **C** Venn diagram showing the overlap between genes with promoters bound by H3K4me3 (*n* = 14,482), genes with promoters bound by MLL1 (*n* = 7097), and differentially expressed genes in the RNA sequencing (RNA-seq) data (*n* = 798). **D** KEGG analyses performed using the 342 intersection genes. **E** Heatmap of downregulated intersection genes that are related to the Hippo signaling pathway according to the RNA-seq data. **F** Western blots of TEAD4 in MLL1-downregulated or MLL1-upregulated BeWo cells. **G** Genome browser view of CUT&Tag signals and RNA-seq tracks for TEAD4 in BeWo cells. **H** The expression levels of *MLL1* and *TEAD4* mRNA correlate positively in multiple human organs according to the GTEx database. **I-J** Quantitative ChIP analysis of H3K4me3 at the *TEAD4* promoter in FSK-treated or *MLL1*-knockdown BeWo cells. The values are normalized to IgG. **K** Quantitative ChIP analysis of MLL1 at the *TEAD4* promoter in FSK-treated BeWo cells. The values are normalized to IgG. **L** Western blots of MLL1, TEAD4, and HCG in BeWo cells exposed to si-MLL1, with or without *TEAD4* overexpression. Data are presented as the means ± SD. **P < 0.01, *P < 0.05. TEAD4, TEA domain transcription factor 4; ChIP, chromatin immunoprecipitation

MLL1 is an important methyltransferase that can activate gene transcription through catalyzing H3K4me3. To identify the exact role of MLL1 and H3K4me3 during syncytialization, we conducted a CUT&Tag experiment for MLL1 and H3K4me3 in BeWo cells. Consistent with previous studies, both H3K4me3 and MLL1 were mainly localized at the promoters of genes (H3K4me3 peaks: n = 14,482, 40.0% at promoters; MLL1 peaks: *n* = 7097, 41.2% at promoters) (Fig. 5A). About 93% (*n* = 6605) of MLL1-targeted genes were also H3K4me3 modified. From the genomic browser of CUT&Tag signals, MLL1 typical target genes reported in other cell types (e.g. *MEIS1*, *PAN3* (encoding Poly(A) specific ribonuclease subunit PAN3), *TP53*, and *PBX3*(encoding PBX homeobox 3)), were also bound by MLL1 in BeWo cells (Fig. 5B and Additional file 2: Fig. S5A). Notably, all these genes had H3K4me3 modifications at their promoter, with peaks similar to the pattern and location of MLL1. Based on these results, we speculated that MLL1 regulates the transcription of specific genes during syncytialization via H3K4me3 modification.

To find the direct MLL1-H3K4me3 target genes regulating the syncytialization process, we intersected the RNA-seq data with genes bound by MLL1 and genes bound by H3K4me3, which identified 342 potential MLL1-H3K4me3 direct target genes (Fig. 5C). After performing a KEGG pathway analysis, surprisingly, we found that the Hippo signaling pathway also gained a high score in the enrichment of these 342 genes (Fig. 5D). Among the 4 down-regulated genes belonging to the Hippo signaling pathway, TEAD4 attracted our attention because it is an important transcription factor responsible for regulating the transcription of downstream genes of the Hippo signaling pathway (Fig. 5E). More importantly, studies have reported that TEAD4 is related to the self-renewal and stemness of trophoblasts [40, 41]. Indeed, both MLL1 and H3K4me3 bound to the promoter of *TEAD4*, and the RNA-seq data revealed a 48.9% reduction in *TEAD4* expression in the si-MLL1 group (Fig. 5F). *MLL1* knockdown or MI-3454 treatment decreased TEAD4 protein levels in BeWo cells, while *MLL1* overexpression increased the level of TEAD4 (Fig. 5G). According to analysis at the GTEx database, *MLL1* and *TEAD4* mRNA expression correlated positively in multiple tissues (Fig. 5H). Therefore, we suggested that *TEAD4* was likely to be the potential direct target gene for MLL1-H3K4me3 during cytotrophoblast differentiation.

Through ChIP assays, we confirmed that *TEAD4* was a direct target gene of MLL1 and H3K4me3 in cytotrophoblast cells (Additional file 2: Fig. S5C-D). We further changed the expression of MLL1 in BeWo cells (Additional file 2: Fig. S5E) and investigated the H3K4me3 and MLL1 levels at the *TEAD4* promoter using four pairs of specific primers (Additional file 2: Fig. S5B). In BeWo cells, FSK treatment reduced H3K4me3 (Fig. 5I) and MLL1 (Fig. 5K) levels at the promoter region of *TEAD4*, and knockdown of *MLL1* also significantly decreased H3K4me3 levels (Fig. 5J).

To determine the exact role of TEAD4 in syncytialization, we induced the time-dependent differentiation of BeWo cells using FSK. The results showed that TEAD4 expression was downregulated after 24 h of FSK-exposure and gradually decreased after 72 h (Additional file 2: Fig. S6A). Overexpression of *TEAD4* significantly decreased HCG, Syncytin 1, and GCM1 levels (Additional file 2: Fig. S6E-H), as well as the syncytium formation (Additional file 2: Fig. S6I) induced by FSK in BeWo cells compared with cells without TEAD4 treatment. Furthermore, *TEAD4* overexpression significantly reversed the si-MLL1-mediated increase in the expression of STB markers (Fig. 5L and Additional file 2: Fig. S6B-D) and blocked si-MLL1-induced cytotrophoblast differentiation (Additional file 2: Fig. S6J).

### Hypoxia inhibited trophoblast syncytialization by regulating the MLL1/ TEAD4 axis

**Fig. 6:**
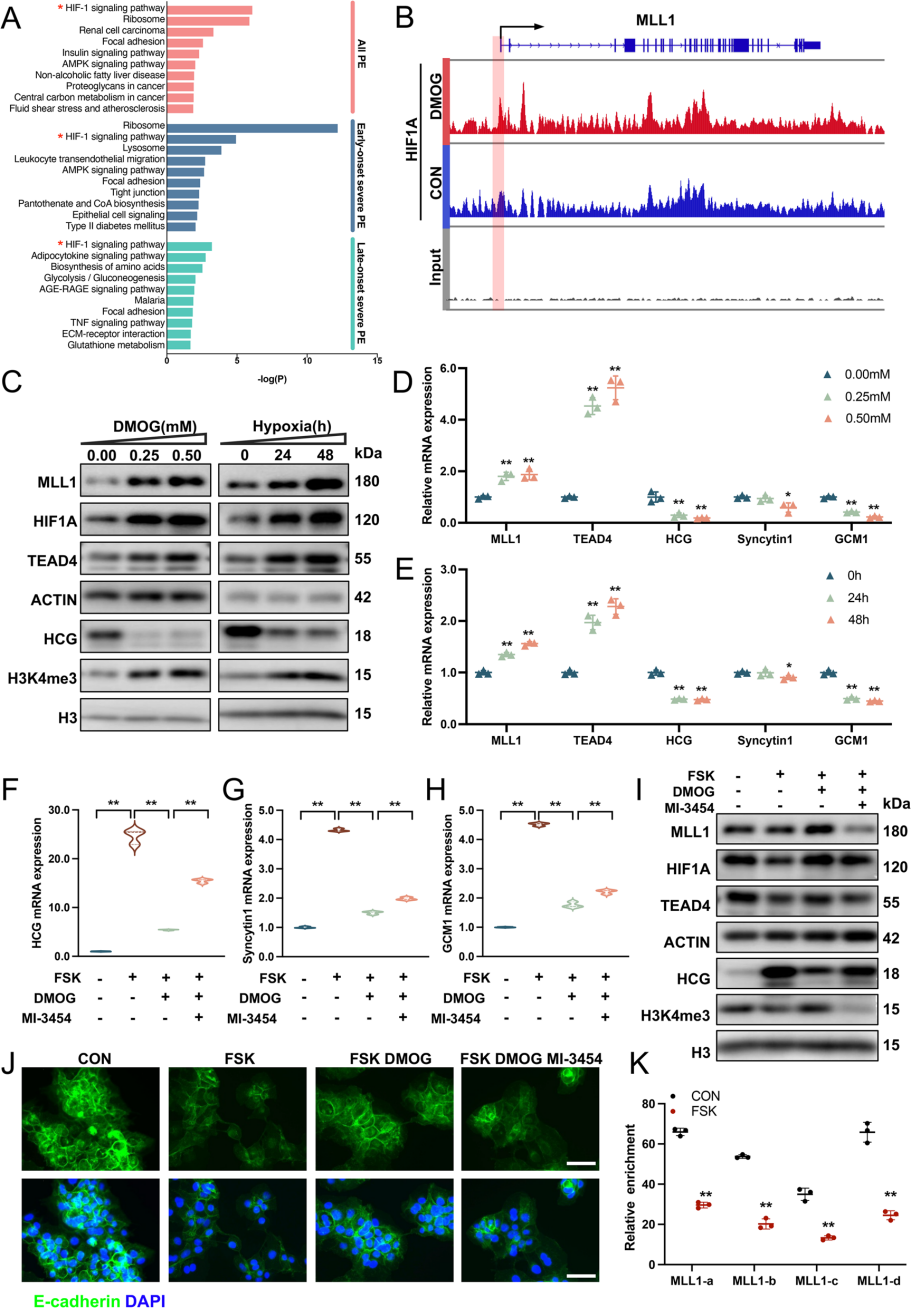
HIF1A regulates trophoblast syncytialization through the MLL1/H3K4me3/TEAD4 axis. **A** KEGG analyses performed on women at different stages of PE and healthy controls. **B** HIF1A chromatin immunoprecipitation sequencing (ChIP-seq) signals for MLL1 in cells treated with DMOG or DMSO. **C** Western blots of MLL1, HIF1A, TEAD4, HCG, and H3K4me3 in BeWo cells treated with a gradient of DMOG concentrations (0, 0.25, and 0.50 mM) and hypoxia exposure time (0, 24, and 48 h). **D** and **E** mRNA levels of *MLL1*, *TEAD4*, and STB markers in BeWo cells treated with a gradient of DMOG concentrations and hypoxia exposure time. **F-J** mRNA levels of STB markers (**F–H**), western blots of MLL1, HIF1A, TEAD4, HCG, and H3K4me3 (**I**) and immunostainings of E-cadherin (green) and DAPI (blue) (**J**) in BeWo cells treated with FSK (25 μM) in the absence or presence of DMOG (0.25 mM) and MI-3454 (5 μM). Scale bar, 40 μm. **K** Quantitative ChIP analysis of HIF1A at the *MLL1* promoter in FSK-treated BeWo cells. The values are normalized to IgG. Data are presented as the means ± SD. **P < 0.01, *P < 0.05. DMOG, dimethyloxallyl glycine; HIF1A, hypoxia-inducible factor 1A

Numerous studies have confirmed a close relationship between tissue hypoxia and the occurrence of syncytialization dysregulation-related diseases, such as PE and IUGR [42–45]. According to the RNA-seq data from Ren et al.[46, 47], KEGG analyses indicated a significant activation of the HIF-1 signaling pathway in early, late, and all stages of PE placenta samples (Fig. 6A). To investigate the role of HIF1A in trophoblast differentiation, we analyzed ChIP-seq data from GSE69100 [48, 49], and found significant HIF1A enrichment in the promoter region of *MLL1*. Dimethyloxallyl glycine (DMOG; MedChemExpress, HY-15893), a low-toxicity compound often employed to simulate hypoxia in cells and tissues, further enhanced this enrichment (Fig. 6B). In addition, we observed a strong positive correlation between the RNA levels of *HIF1A* and *MLL1* across multiple organs, with a correlation coefficient of 0.75 in whole blood (Additional file 2: Fig. S5F). These findings suggested that HIF1A might be an important upstream regulator of MLL1, potentially contributing to the pathogenesis of PE.

Stimulation with a gradient of DMOG concentrations (0, 0.25, and 0.50 mM) and hypoxia exposure time (0, 24, and 48 h) led to a gradual increase in the protein level of HIF1A, accompanied by the upregulation of MLL1 and TEAD4 expression, an increase in the H3K4me3 level, and a decrease in the HCG level (Fig. 6C). In the qRT-PCR experiment, as HIF1A gradually accumulated, we also observed a gradient increase in *MLL1* and *TEAD4* expression and a decreasing trend in the expression of STB markers (Fig. 6D and E). Moreover, a rescue experiment further demonstrated the regulatory relationship between HIF1A and MLL1. The results showed that the upregulation of HIF1A protein levels inhibited FSK-mediated trophoblast syncytialization. However, this phenomenon could be rescued by adding the inhibitor MI-3454, leading to an increase in the expression of STB markers (Fig. 6F-I) as well as syncytium formation (Fig. 6J).

To further validate the regulatory role of HIF1A on MLL1 in trophoblasts, a ChIP experiment was conducted. Based on the ChIP-seq results, four pairs of primers were designed in the promoter region of *MLL1* (Additional file 2: Fig. S5G). The results of the ChIP experiment showed significant enrichment of HIF1A in the promoter region of *MLL1* (Additional file 2: Fig. S5H). In addition, compared with that in the control group, the enrichment level in the FSK group decreased significantly (Fig. 6K). These results suggested that HIF1A regulates the transcription of *MLL1* by binding to its promoter region, thereby participating in the process of trophoblast differentiation and placental development.

### Upregulated TEAD4 and HIF1A in placental villous tissue of patients with preeclampsia

**Fig. 7:**
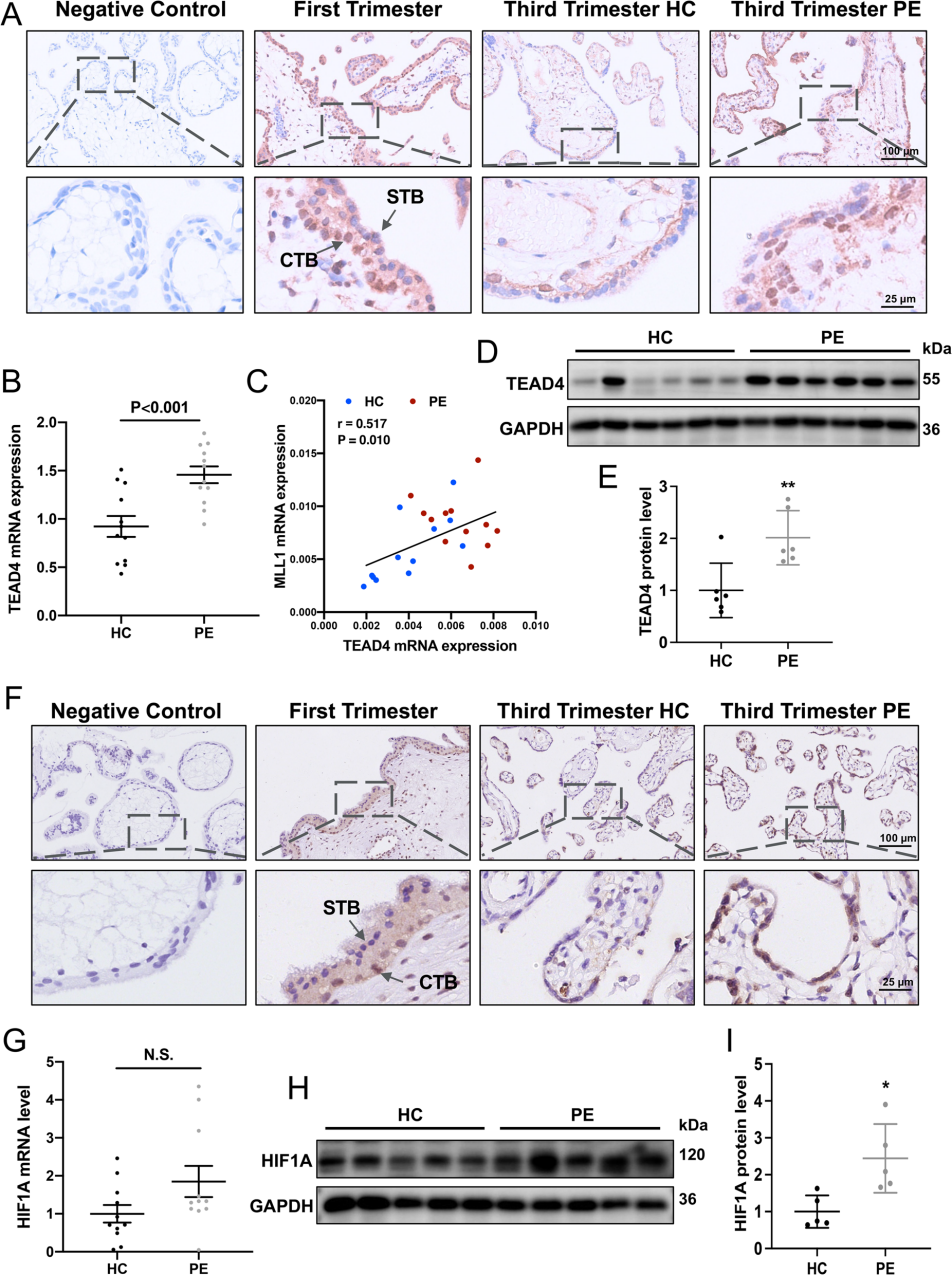
Distribution and levels of TEAD4 and HIF1A in human placental villi. **A and F** Immunohistochemical analysis of TEAD4 (**A**) and HIF1A (**F**) staining in pregnant women during the first trimester (*n* = 6), third trimester (*n* = 6), and preeclampsia (*n* = 6). **B** and **G** mRNA levels of *TEAD4* (HC group, *n* = 12; PE group, *n* = 12) (**B**) and *HIF1A* (HC group, *n* = 11; PE group, *n* = 11) (**G**) in women with preeclampsia and healthy controls. **C** The *TEAD4* mRNA level correlated positively with the mRNA level of *MLL1* in placental villi in PE and HC. **D**, **E**, **H** and **I** Western blots (**D** and **H**) and corresponding quantification (**E** and **I**) of TEAD4 (HC group, *n* = 6; PE group, *n* = 6) and HIF1A (HC group, *n* = 5; PE group, *n* = 5) in the PE and control women. Data are presented as the means ± SD. **P < 0.01, *P < 0.05

Immunohistochemical staining showed that TEAD4 was localized primarily in the nuclei of cytotrophoblasts at early gestation, and the staining of TEAD4 in the PE group was more intense than that in the HC group (Fig. 7A). Moreover, according to the qRT-PCR and western blotting analyses, women with PE had higher levels of TEAD4 than did the HCs (Fig. 7B, D-E). The mRNA expression of *MLL1* and *TEAD4* correlated positively in villous tissues (r = 0.517 and *P*-value = 0.010) (Fig. 7C). Likewise, HIF1A was mainly detected in the nuclei of cytotrophoblasts during early gestation, and its staining intensity was significantly higher in the PE group than in the HC group (Fig. 7F). The protein level of HIF1A was significantly elevated in the PE group (Fig. 7H and I), while there was no significant difference in the RNA level (Fig. 7G). In summary, these results suggested that the villous trophoblasts from PE pregnancies are characterized by increased expression of HIF1A and TEAD4, a finding that is consistent with the results of in vitro experiments conducted in BeWo cells.

### Mll1 inhibition in pregnant mice promotes trophoblast syncytialization and placental growth

**Fig. 8:**
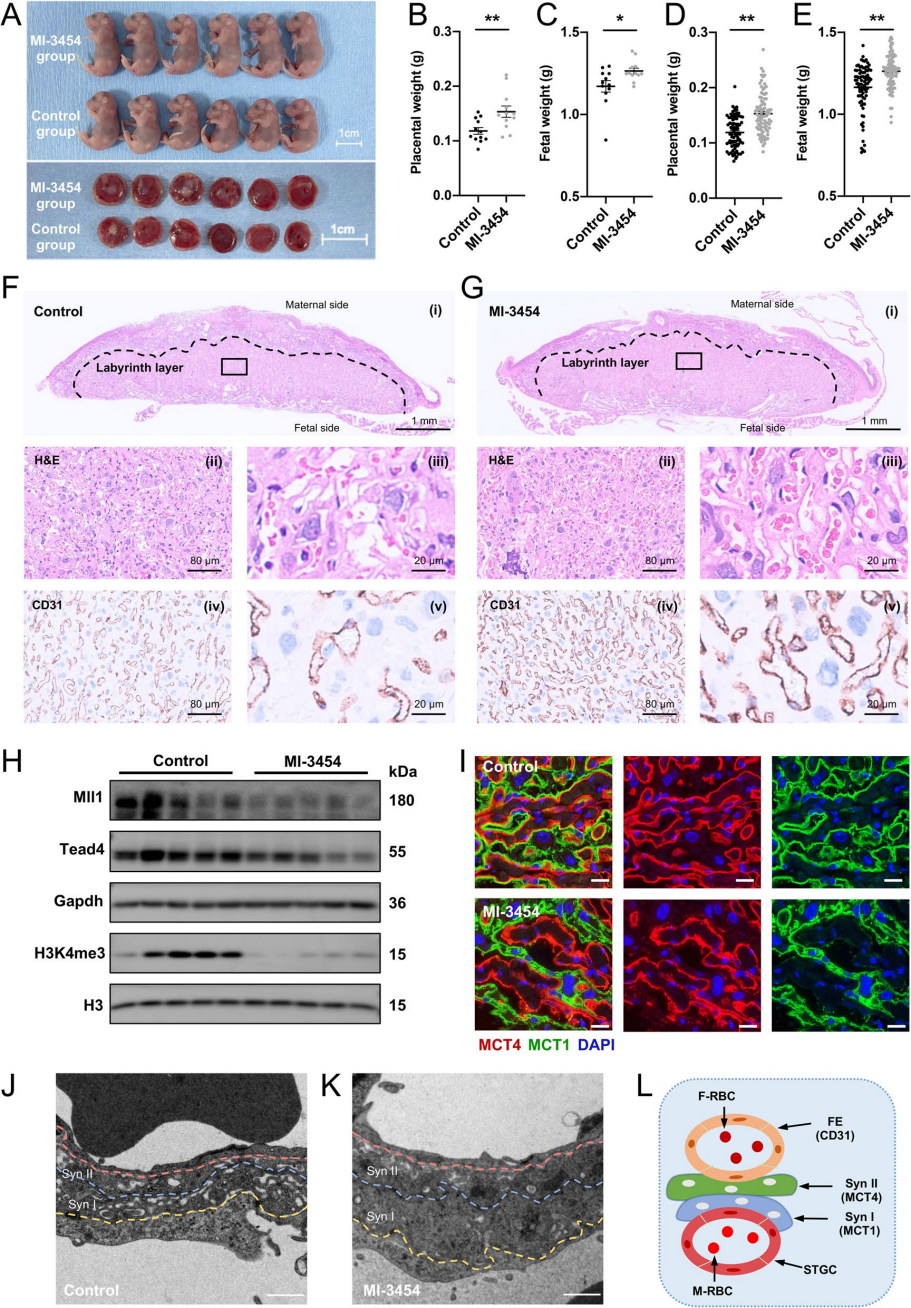
Effect of intraperitoneal administration of MI-3454 on fetoplacental weights in mice. **A** Representative appearance of the MI-3454 and control fetuses and placentas. **B-E** Administration of inhibitor MI-3454 (15 mg/kg boy weight (BW)) increased the average fetal and placental weights both litters (Control group, *n* = 12; MI-3454 group, *n* = 12) (**B** and **C**) or in fetuses (Control group, *n* = 87; MI-3454 group, *n* = 88) (**D** and **E**). **F** and **G** H&E staining (i-iii) and immunostaining against CD31 (iv and v) of the control (*n* = 6) (**F**) and MI-3454 (*n* = 6) (**G**) placentas at E18.5. Panels (iii) and (v) in F and G are the corresponding higher magnification images of panels (ii) and (iv), respectively. (H) Western blots of Mll1, Tead4, and H3K4me3 in the control (*n* = 5) (F) and the MI-3454 (*n* = 5) (G) placentas at E18.5. (I) Immunofluorescent staining of MCT1 (green), MCT4 (red), and DAPI (blue) in the indicated placentas (*n* = 5). Scale bar, 20 μm. (J and K) Representative transmission electron microscopy images of STBs in mouse placentas treated with vehicle (*n* = 3) (J) or MI-3454 (*n* = 3) (**K**). Scale bar, 1 μm. **L** Schematic depiction of the mouse placenta. MCT1 specifically expresses in the SynT-1 layer, while MCT4 specifically stains the SynT-2 layer. Data are presented as the means ± SD. **P < 0.01, *P < 0.05. FE, fetal endothelium; F-RBC, fetal red blood cell; M-RBC, maternal red blood cell; STGC, sinusoidal trophoblast giant cell; Syn I, the first layer of STBs; Syn II, the second layer of STBs; H&E, hematoxylin and eosin; CD31, platelet and endothelial cell adhesion molecule 1; MCT1, monocarboxylate transporter 1; MCT4, monocarboxylate transporter 4

To explore the function of Mll1 in placental and fetal growth in vivo, intraperitoneal injection of MI-3454 was conducted from E12.5 to E17.5 and the fetoplacental development was examined on E18.5 (Additional file 2: Fig. S7A). The placentas and fetuses of the MI-3454 group were larger than those of the control group (Fig. 8A). MI-3454 administration significantly increased the average placental and fetal weights in the litter (Fig. 8B and C) or the fetus (Fig. 8D and E). Moreover, we found that Mll1, Tead4, and H3K4me3 levels were significantly decreased following MI-3454 treatment, consistent with our results in cultured human trophoblasts (Fig. 8H). The mRNA levels of STB markers, *Gcm1*, Syncytin-a (*Syna*), and Syncytin-b (*Synb*) were significantly upregulated in the MI-3454 group (Additional file 2: Fig. S7B-D), suggesting elevated syncytialization upon MI-3454 treatment in pregnant mice.

H&E staining of placentas in the MI-3454 group revealed that their labyrinth layer was markedly thicker than that in the controls [Fig. 8F (i), G (i) and Additional file 2: Fig. S7E]. In comparison with the control group, the labyrinths of the placentas in the MI-3454 group were better packed, with more fetal blood vessels containing erythrocytes [Fig. 8F (ii, iii) and G (ii, iii)]. The fetal blood vessels were further labeled with CD31. The increase in CD31 staining indicated that the fetal vessels in the placentas in the MI-3454 group were able to develop and branch better in the labyrinth layer [Fig. 8F (iv, v) and G (iv, v)]. To evaluate the syncytiotrophoblast formation of placentas, two syncytiotrophoblast layers, SynT-1 and SynT-2, were stained using MCT1 and MCT4, respectively (Fig. 8L). The results showed a remarkably altered alignment of the syncytiotrophoblast in the placentas in the MI-3454 group, reflected by an expanded separation between the MCT1 and MCT4 layers (Fig. 8I). We further observed the placenta using transmission electron microscopy to detect the delicate structure of the STB layers. Phenotypic changes consistent with immunofluorescence results were found in the placentas of the MI-3454 group, as evidenced by a marked thickening of the syncytial layer (Fig. 8J and K). All these morphological results indicated enhanced syncytialization and placental growth upon MI-3454 treatment in pregnant mice.

## Discussion

**Fig. 9:**
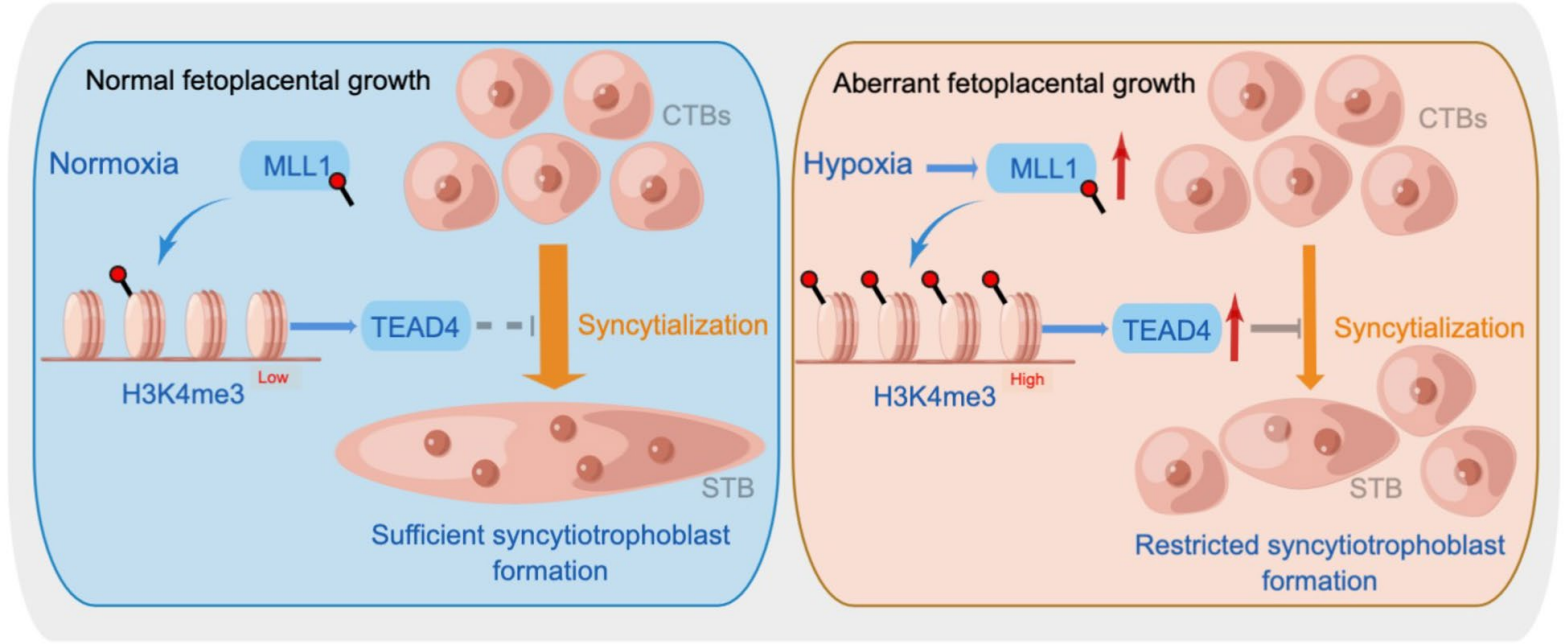
Diagram depicting the role of MLL1 in the process of trophoblast syncytialization. The expression of MLL1 shows a negative correlation with syncytialization

Normal trophoblast syncytialization is the foundation for placental development and the health protection for both mothers and infants. However, the process by which cytotrophoblasts differentiate into syncytiotrophoblasts is complicated, and there has been limited exploration of its mechanisms. To date, epigenetic mechanisms have been rarely linked to syncytialization. The current study used a library of epigenetic compounds for forward drug screening, connected epigenetic regulation with syncytialization, and provided new insights to determine the function of MLL1 in regulating placental and fetal growth (Fig. 9).

MLL1, a histone methyltransferase, is responsible for catalyzing the H3K4me3 mark at gene promoters and regulates the expression of target genes, such as *MEIS1*, *TP53*, and *PBX3* [50–53]. A dramatic whole genomic chromatin alteration, characterized by chromatin accessibility and histone modification, was observed during human trophoblast differentiation [54]. However, the effect of the H3K4me3 modification in the syncytial layer is uncertain. Unique histone methyltransferases in large COMPASS (Complex Proteins Associated with Set1)-like complexes modulate histone modifications in different types of cells [55]. In mammals, there are six homologs (SET domain containing 1A (SET1A), SET1B,and MLL1–4) executing the catalytic function of H3K4 methylation [56]. Among them, the SET1A/B family mediates the H3K4me2/H3K4me3 modification and the MLL3/4 family regulates the H3K4me1 modification. The MLL1/2 family is responsible for the H3K4me3 modification at the promoter region and transcription start site of various genes [55]. In this study, we demonstrated the H3K4me3 profiling during trophoblast syncytialization and revealed the mechanism of its regulation by MLL1.

Among the direct targets marked by MLL1-H3K4me3, TEAD4 is involved in the regulation of syncytialization, encoding a core transcription factor of the Hippo signaling pathway. Previous studies highlighted the Hippo signaling pathway's crucial role in development, cell fate, stemness, and differentiation [38, 57–59]. Activation of the Hippo signaling pathway involves a sequence of phosphorylation events, ultimately inhibiting Yes-associated protein (YAP) and transcriptional coactivator with PDZ-binding motif (TAZ) [57]. Conversely, when the Hippo pathway is inactive, unphosphorylated YAP/TAZ move to the nucleus, where they enhance stemness and proliferation as coactivators of the TEAD transcription factor family [60]. The TEAD family is widely involved in various physiological processes, including embryonic stem cell differentiation, trophoblast stem cell differentiation, embryo implantation, and fetoplacental growth because of its powerful role in regulating gene transcription. A previous study found that TEAD4 directly modulates cyclin dependent kinase 1 (CDK1) transcription and a series of genes related to the cell cycle [40]. In TEAD4-depleted trophoblast cells, dysregulation of cell cycle regulators could further lead to deficient cell proliferation, suggesting that TEAD4 is a vital regulator that maintains trophoblast self-renewal. Besides activating the cell cycle, TEAD4 can maintain trophoblast stem cell or CTB stemness by binding to the promoter sequences of STB marker genes such as *CGB* (encoding chorionic gonadotropin subunit beta) and *ERVW-1* [35, 40]. The formation of the YAP–TEAD4–enhancer of zeste 2 (EZH2) complex silences their transcription and thereby suppresses cell fusion [35]. These are the potential mechanisms by which TEAD4 regulates the syncytialization process. In addition, we observed that knocking down *MLL1* led to alterations in the expression of a series of genes related to the Hippo signaling pathway, including *MOB1A*, *TEAD1, TEAD3*, and *TEAD4*. Therefore, besides directly regulating TEAD4 transcription, MLL1 might also have a broad impact on the expression of other genes in the Hippo signaling pathway through indirect mechanisms, collectively influencing syncytialization. Further research is needed to investigate this aspect.

Hypoxia might not be the sole upstream regulator of the MLL1-H3K4me3 axis. Pathways such as Inhibitor of nuclear factor kappa-B kinase (IKK)/nuclear factor kappa B (NF- κB) signaling might also regulate MLL1 protein retention and the necessary histone modifications for the transcription of crucial target genes [61]. Besides, in lung fibroblast cells, the damaged DNA binding proteins, damage specific DNA binding protein 1 (DDB1) and cullin 4 (CUL4), can regulate MLL1-mediated H3K4 methylation by recruiting diverse WD40 proteins and forming different E3 ubiquitin ligases [62]. Therefore, further research is necessary to investigate whether other upstream factors exist that regulate the expression of MLL1 and their exact roles in syncytialization.

Abnormal syncytialization has been associated with various human diseases, such as IUGR and PE, highlighting the potential therapeutic benefit of interfering with this process. MLL1 plays an essential role in syncytialization; therefore, our research establishes a mechanistic basis for assessing the therapeutic potential of MLL1 inhibitors to treat syncytialization dysregulation-related diseases. Decreased syncytiotrophoblast formation and increased syncytiotrophoblast apoptosis lead to elevated levels of syncytiotrophoblast-derived microparticles (STBMs), which cannot be effectively cleared by macrophages and are transferred into the maternal circulation [63, 64], contributing to the development of PE. Therefore, MI-3454 might ameliorate conditions like PE by elevating syncytialization levels, thereby inhibiting the production of pathogenic factors. Many MLL1 inhibitors have already shown potential to treat acute leukemia, including acute myeloid leukemia (AML) and acute lymphoblastic leukemia [65]. For example, the FDA has granted orphan drug designation to SNDX-5613 for the treatment of refractory/relapsed leukemia, and to KO-539 for the treatment of AML [65]. Notably, KO-539 is a structural analog of MI-3454, which has demonstrated the ability to induce complete remissions in two patients with AML and showed activity in other participants in a phase I trial [66]. MLL1 inhibitors have been shown to be effective and safe in treating acute leukemia, and according to our research, it is possible that they could also be used to promote syncytialization, consequently offering the potential to improve the outcomes of various syncytialization dysregulation-associated diseases.

It is worth noting that the translation of theoretical discoveries into practical applications is a multifaceted process. Despite having established some potential benefits, it is paramount to acknowledge that the use of MLL1 inhibitors during pregnancy might also carry inherent risks. These risks necessitate comprehensive evaluation through stringent clinical trials, commencing with a thorough examination of their toxicological profiles. Furthermore, a meticulous inquiry into optimal dosage, administration routes, and timing is imperative to mitigate any potential adverse effects on both maternal and fetal development. Our study is primarily aimed at uncovering novel insights into the mechanisms underlying syncytialization dysregulation-related diseases and endeavoring to propose potential therapeutic strategies. The practical implementation of these findings requires further extensive research and validation in the future.

The BeWo cell line is a valuable model to investigate trophoblast syncytialization, which has been widely employed in previous research for this purpose [8, 67–69]. However, it is important to recognize that BeWo cells might undergo changes in gene expression under in vitro culture conditions, differing from the physiological state of primary cells. As a result, these cells may not necessarily fully reflect the properties of healthy human trophoblasts. In this regard, to enhance the accuracy and reliability of our experimental findings, our conclusions were validated using another trophoblast cell line, JEG3, which also exhibited the ability to undergo syncytialization in vitro [35, 36]. The confirmation of our findings across diverse cell lines provides additional support and reinforces the robustness and reliability of the conclusions drawn in this study. One limitation of this study is that, because of experimental constraints and challenges in specimen collection, experiments involving the isolation of primary human trophoblast were not undertaken.

## Conclusions

We demonstrated that MLL1-H3K4me3 plays a vital role in syncytialization progression by regulating the transcription of *TEAD4*. Moreover, HIF1A exerted regulatory control over this process. These findings emphasized the diagnostic and therapeutic possibilities of targeting MLL1 for syncytialization-associated diseases and revealed a direct correlation between the epigenetic modulation of histone modification and fetoplacental growth.

### Supplementary Information

**Additional file 1: Table S1.** Details of the epigenetic compound library.

**Additional file 2: Fig. S2.** Further validation of the functional properties of the top 10 epigenetic drugs. **Fig. S2.** Inhibition of MLL1 promoted syncytialization. **Fig. S3.** Overexpression of MLL1 Inhibited FSK-Induced syncytialization. **Fig. S4.** JEG3 cell line is employed for further validation in in vitro experiments. **Fig. S5.** TEAD4 is a direct target gene of MLL1 and MLL1 is a direct target gene of HIF1A in BeWo cells. **Fig. S6.** The function of TEAD4 in the process of syncytialization. **Fig. S7.** Mll1 inhibition in pregnant mice promotes trophoblast syncytialization. **Table S2.** Human primer Information. **Table S3.** Mouse primer information.

**Additional file 3. **Original blot images.

## Data Availability

The datasets supporting the conclusions of this article are available in the NCBI Sequence Read Archive database (accession numbers PRJNA975296 and PRJNA975314).

## Abbreviations

MLL1: Mixed lineage leukemia 1
TEAD4: TEA domain transcription factor 4
FSK: Forskolin
HIF1A: Hypoxia-inducible factor 1A
IUGR: Intrauterine growth restriction
PE: Preeclampsia
CTBs: Cytotrophoblasts
STBs: Syncytiotrophoblasts
HMTs: Histone methyltransferases
IPMCH: International Peace Maternity and Child Health Hospital
DMEM: Dulbecco’s modified Eagle’s medium
DMSO: Dimethyl sulfoxide
PPI: Protein–protein interaction
GCM1: Glial cells missing transcription factor 1
GAPDH: Glyceraldehyde-3-phosphate dehydrogenase
H3: Histone 3
H&E: Hematoxylin and eosin
MCT1: Monocarboxylate transporter 1
TEM: Transmission electron microscopy
IHC: Immunohistochemical
DEGs: Differentially expressed genes
DMOG: Dimethyloxallyl glycine
Syna: Syncytin-a
Synb: Syncytin-b
YAP: Yes-associated protein
TAZ: Transcriptional coactivator with PDZ-binding motif
CDK1: Cyclin dependent kinase 1
AML: Acute myeloid leukemia
