# Supplementary material for: Epigenetic drug screening for trophoblast syncytialization reveals a novel role for MLL1 in regulating fetoplacental growth
Source: BMC Med. 2024 Feb 5;22:57. doi: 10.1186/s12916-024-03264-8 (PMC10845764; doi:10.1186/s12916-024-03264-8)
Supplement: Supplementary file 2 — Additional file 2: Fig. S2. Further validation of the functional properties of the top 10 epigenetic drugs. Fig. S2. Inhibition of MLL1 promoted syncytialization. Fig. S3. Overexpression of MLL1 Inhibited FSK-Induced syncytialization. Fig. S4. JEG3 cell line is employed for further validation in in vitro experiments. Fig. S5. TEAD4 is a direct target gene of MLL1 and MLL1 is a direct target gene of HIF1A in BeWo cells. Fig. S6. The function of TEAD4 in the process of syncytialization. Fig. S7. Mll1 inhibition in pregnant mice promotes trophoblast syncytialization. Table S2. Human primer Information. Table S3. Mouse primer information. [file 12916_2024_3264_MOESM2_ESM.doc]

Fig. S1

**
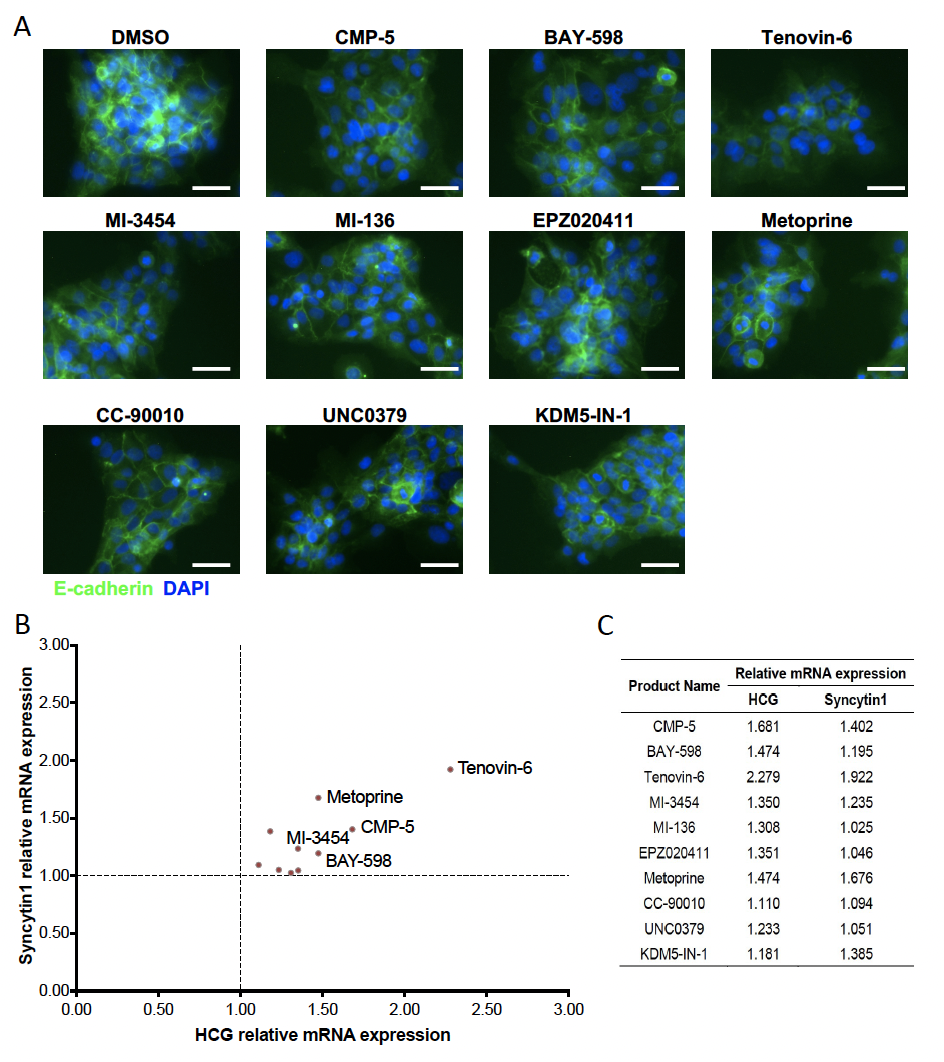
**

**Fig. S1 Further validation of the functional properties of the top 10 epigenetic drugs.** (A) The top 10 epigenetic drugs effectively promoted the fusion of BeWo cells. Scale bar, 40 μm. (B) Scatter plot displaying the effects of top 10 epigenetic drugs on the syncytialization process of BeWo cells along with the FSK treatment (25 μM). The y-axis and x-axis show the relative mRNA levels of two STB markers after administration of each drug, normalized against the control group exclusively treated with FSK. Each dot represents an individual drug. (C) A list of 10 drugs is shown with their relative mRNA levels of two STB markers.

Fig. S2

**
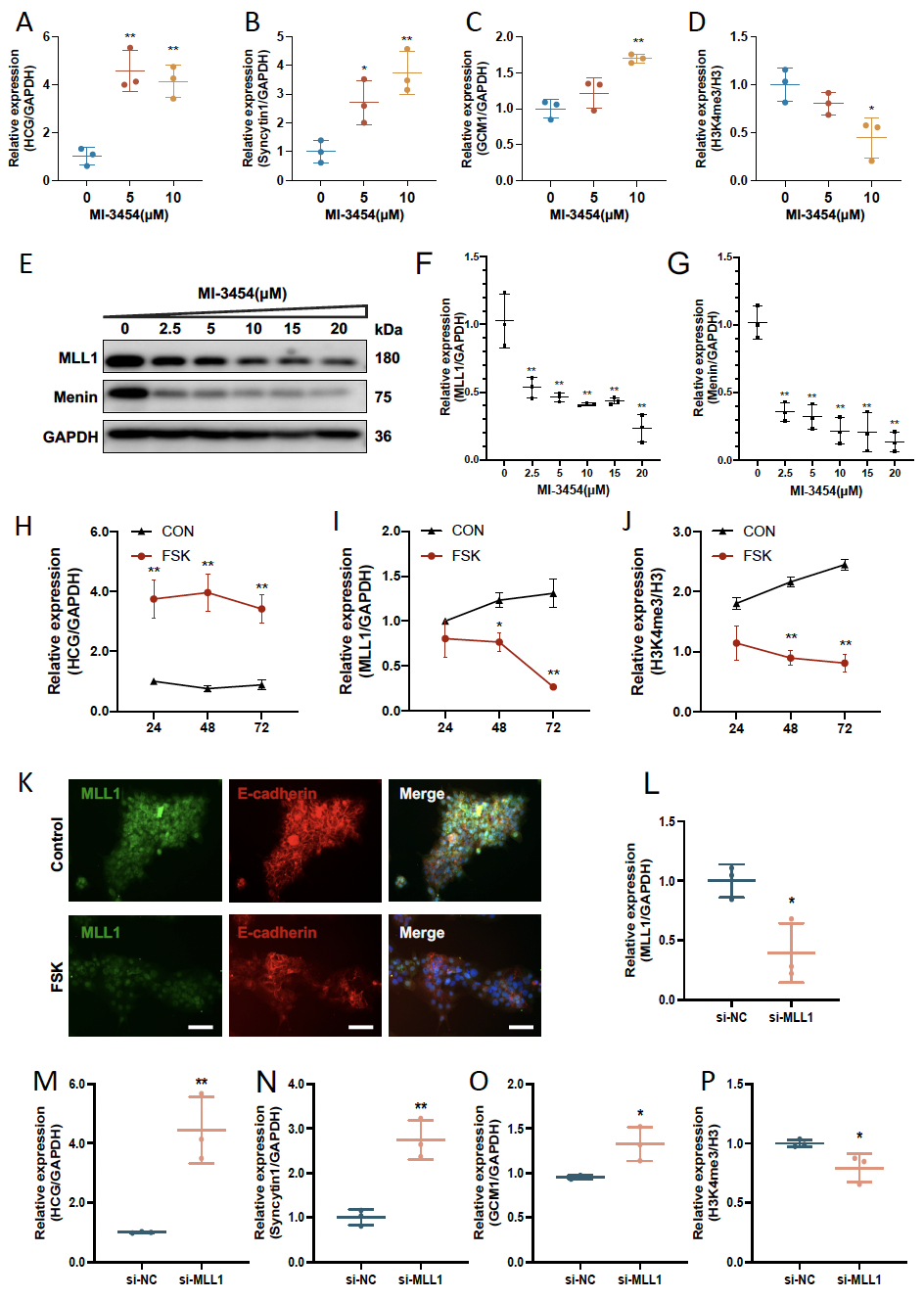
Fig. S2 Inhibition of MLL1 promoted syncytialization.** (A-D) Quantification of western blots for H3K4me3 and STB markers in BeWo cells exposed to 0, 5, and 10 μM MI-3454. (E-G) Western blots (E) and corresponding quantification (F and G) of MLL1 and Menin in BeWo cells treated with 0 to 20 μM MI-3454. (H-J) Quantification of western blots for MLL1, HCG, and H3K4me3 in BeWo cells treated with or without 25μM FSK for 24, 48, and 72 hours. (K) Representative immunostaining of MLL1 (green), E-cadherin (red), and DAPI (blue) in BeWo cells treated with or without 25μM FSK for 48 hours. Scale bar, 40 μm. (L-P) Quantification of western blots for MLL1, H3K4me3, and STB markers in BeWo cells transfected with si-NC and si-MLL1. Data are presented as the means ± SD. **P < 0.01, *P< 0.05.

Fig. S3


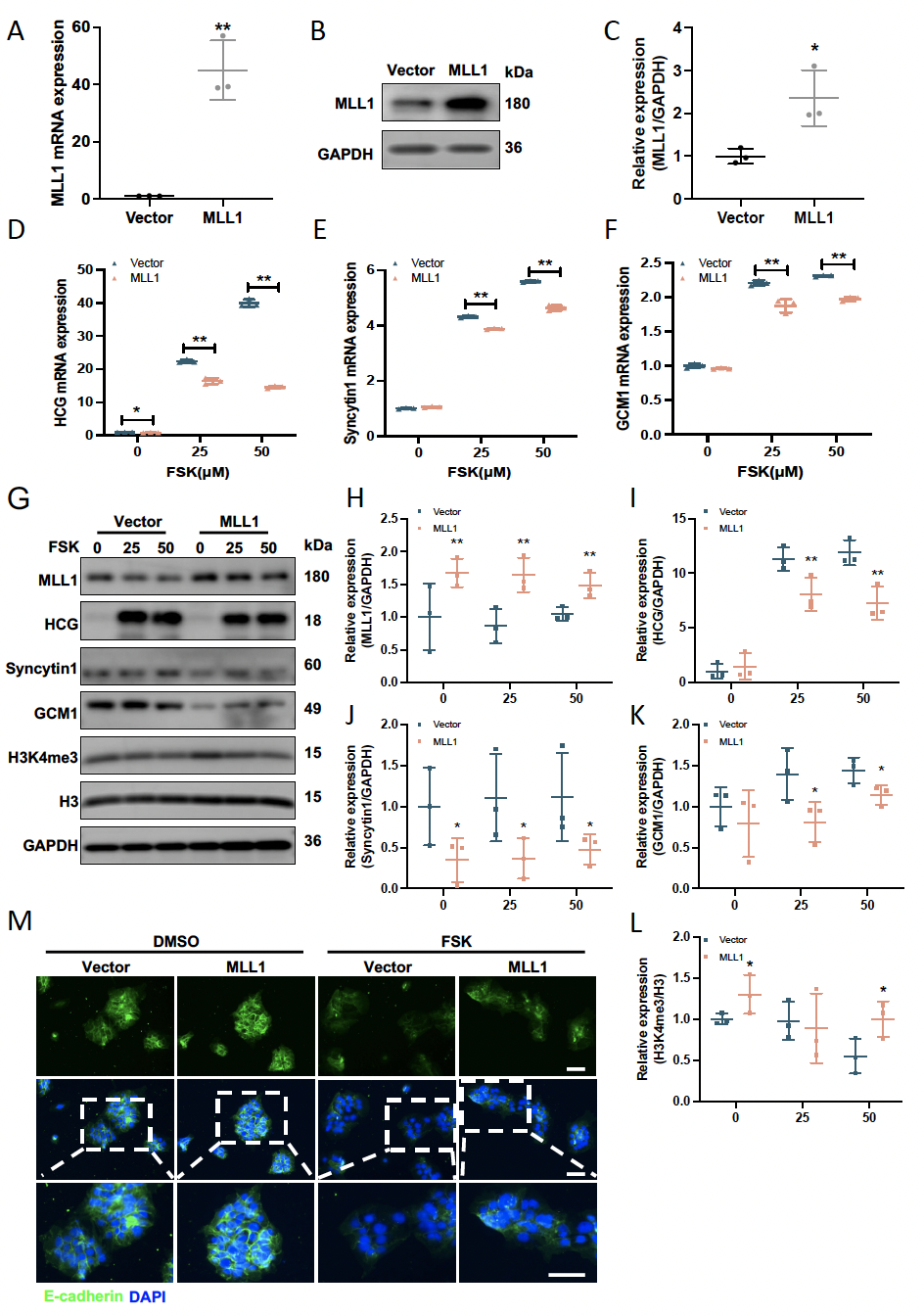


**Fig. S3 Overexpression of MLL1 Inhibited FSK-Induced syncytialization.** (A-C) Efficiency of *MLL1* overexpression identified by qRT-PCR (A) and western blots (B and C). (D-L) mRNA levels of STB markers (D-F), western blots (G) and corresponding quantification (H-L) of MLL1, H3K4me3, and STB markers in BeWo cells transfected with the MLL1 plasmid or empty vector, exposed to 0, 25, and 50 μM FSK. (M) Immunostainings of E-cadherin (green) and DAPI (blue) in BeWo cells transfected with MLL1 plasmid or vector, exposed to DMSO or FSK. Scale bar, 40 μm. Data are presented as the means ± SD. **P < 0.01, *P < 0.05.

Fig. S4

**
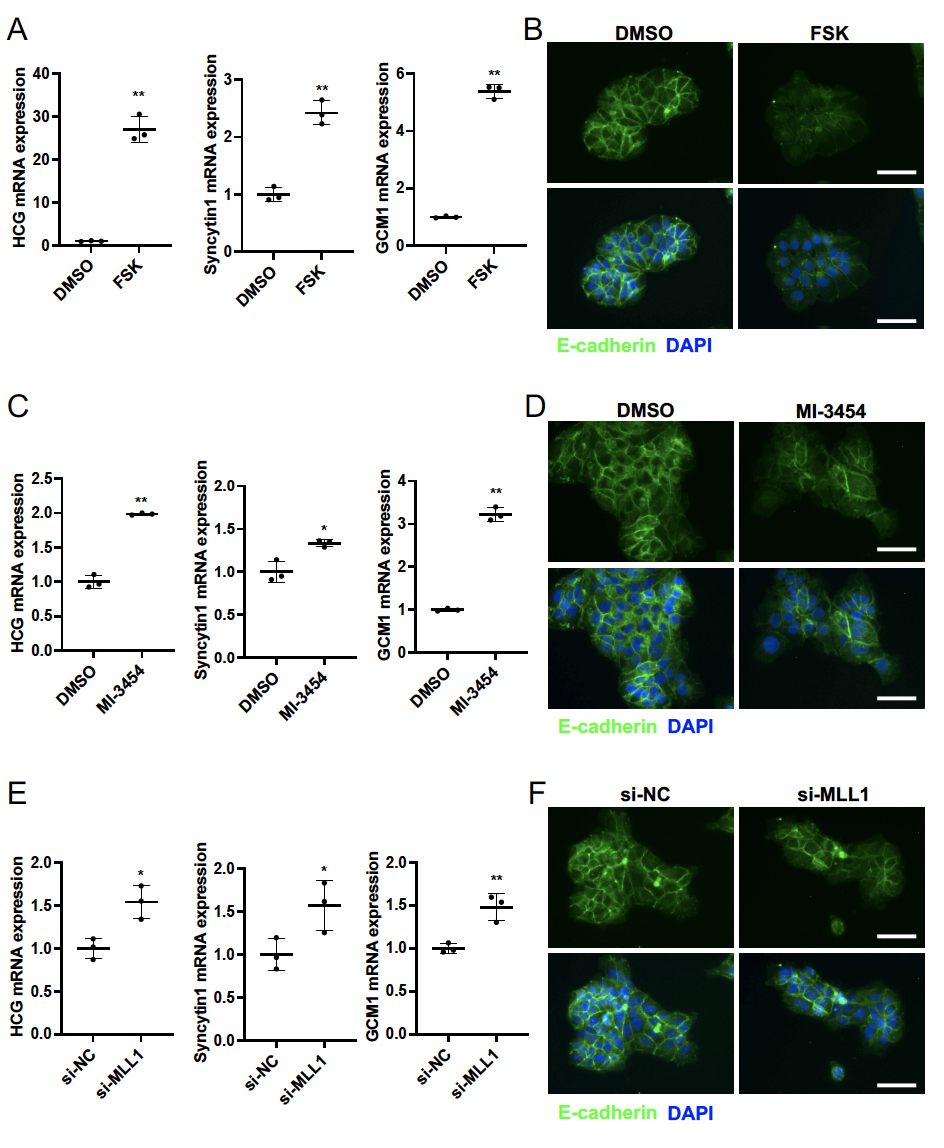
**

**Fig. S4 JEG3 cell line is employed for further validation in in vitro experiments.** (A-B) JEG3 cell line exhibits the ability to undergo syncytialization under FSK induction. (C-F) The MI-3454 treatment (C-D) or the knockdown of MLL1 (E-F) facilitate syncytialization in JEG3 cells. Scale bar, 40 μm. Data are presented as the means ± SD. **P < 0.01, *P < 0.05.

Fig. S5

**
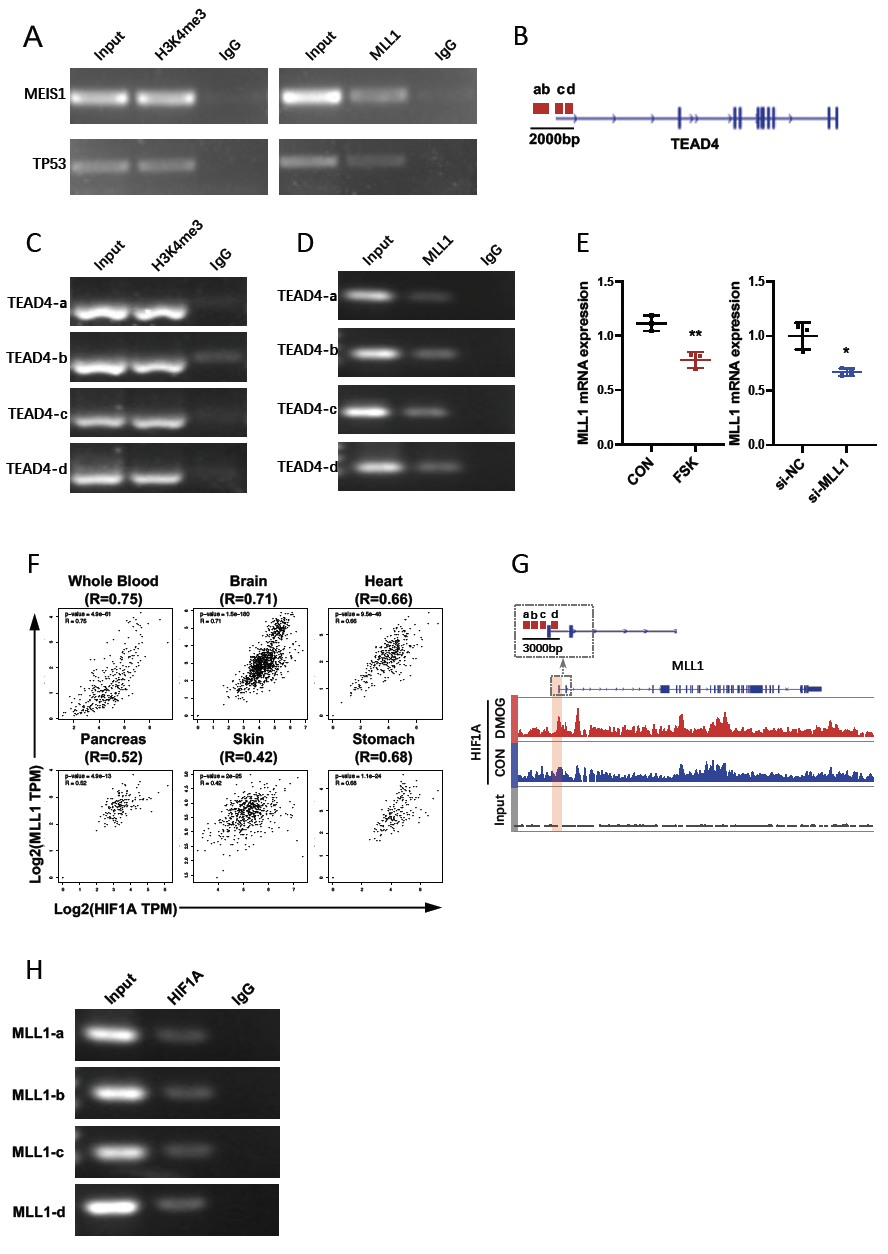
Fig. S5 *TEAD4* is a direct target gene of MLL1 and *MLL1* is a direct target gene of HIF1A in BeWo cells.** (A) ChIP PCR assays for the *MEIS1* and *TP53* promoter. BeWo cells were subjected to ChIP assays using MLL1 and H3K4me3 antibodies. Immunoglobulin G (IgG) served as the control. (B) Four pairs of specific primers were designed in the promoter region of *TEAD4.* (C-D) ChIP PCR assays for the *TEAD4* promoter. BeWo cells were subjected to ChIP assays using MLL1 and H3K4me3 antibodies. IgG served as the control. (E) mRNA levels of *MLL1* in the BeWo cells after FSK treatment or knocking down with specific siRNAs against *MLL1*. (F) The expressions of *HIF1A* and *MLL1* mRNA correlate positively in multiple human organs from the GTEx database. (G) Four pairs of specific primers were designed in the promoter region of *MLL1* according to the ChIP-seq data. (H) ChIP PCR assays for the *MLL1* promoter. BeWo cells were subjected to ChIP assays using anti-HIF1A antibodies. IgG served as the control. Data are presented as the means ± SD. **P < 0.01, *P < 0.05.

Fig. S6


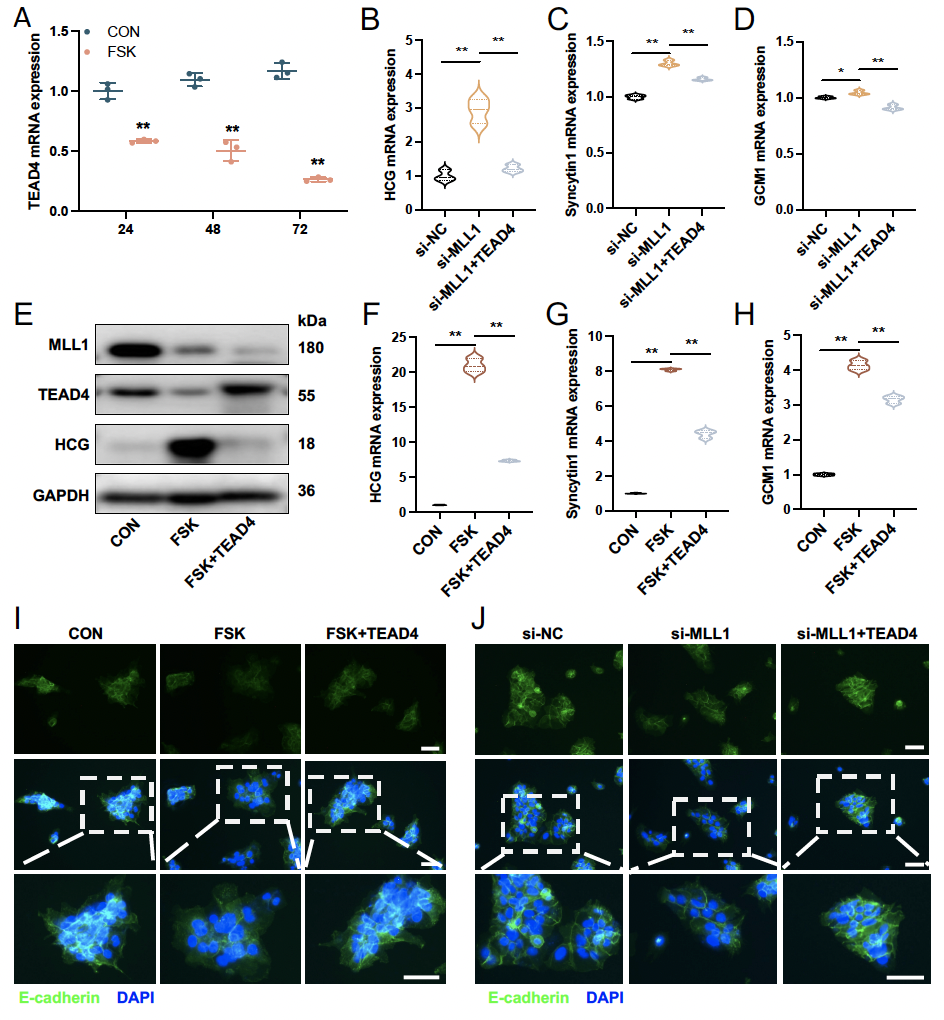
**Fig. S6 The function of TEAD4 in the process of syncytialization.** (A) mRNA levels of TEAD4 in BeWo cells treated with or without 25μM FSK for 24, 48, and 72 hours. (B-D) mRNA levels of STB markers in BeWo cells treated with si-MLL1 in the absence or presence of *TEAD4* overexpression. (E-H) Western blots of MLL1, TEAD4, HCG (E) and mRNA levels of STB markers in BeWo cells treated with FSK (F-H) in the absence or presence of *TEAD4* overexpression. (I-J) Immunostainings of E-cadherin (green) and DAPI (blue) (M and N) in BeWo cells exposed to FSK (I) or si-MLL1 (J), with or without TEAD4 overexpression. Scale bar, 40 μm. Data are presented as the means ± SD. **P < 0.01, *P < 0.05.

Fig.S7
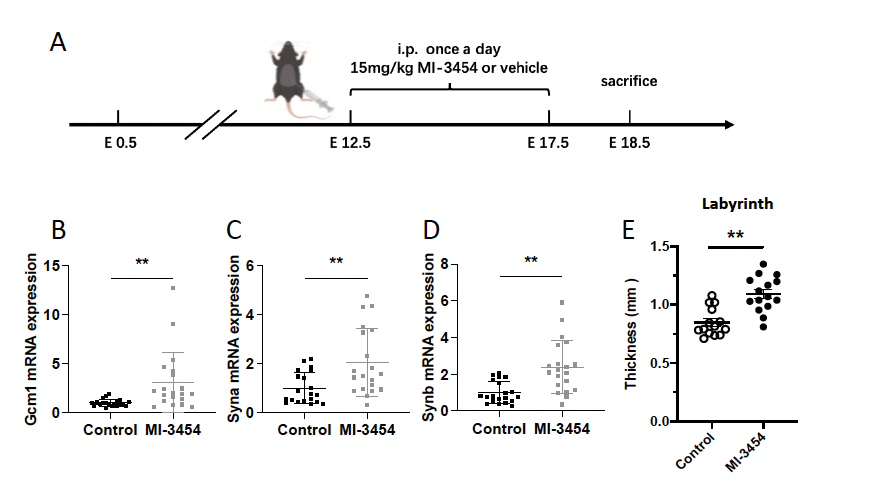


**Fig. S7 Mll1 inhibition in pregnant mice promotes trophoblast syncytialization.** (A) Time-line illustrating experimental procedures in pregnant mice. (B-D) mRNA levels of STB markers in the control (n = 20) and the MI-3454 placentas (n = 20) at E18.5. (E) The lengths of the labyrinth in the center of the placenta were measured in the control (n = 14) and the MI-3454 (n = 15) placentas at E18.5. Data are presented as the means ± SD. **P < 0.01, *P < 0.05. i.p., intraperitoneal injection; TPM, transcript per million.

Table S2. Human primer Information.

| Name | Sequences (5′ to 3′) |
| --- | --- |
| qRT-PCR primers | |
| ACTIN-F | GTCATTCCAAATATGAGATGCGT |
| ACTIN-R | GCTATCACCTCCCCTGTGTG |
| HCG-F | GTCAACACCACCATCTGTGC |
| HCG-R | GCACATTGACAGCTGAGAGC |
| Syncytin 1-F | CTTCCTCTCATTCTTAGTGCCC |
| Syncytin 1-R | CCAATGCCAGTACCTAGTGC |
| GCM1-F | GAGGCAAGAAGAGCCATGAA |
| GCM1-R | TCTGTGATTCCTCCCAGACC |
| MLL1-F | AAGAGCAGGTAAACTCTCTCCTC |
| MLL1-R | TTCCTCTCCGTCGTACAATTTG |
| TEAD4-F | GAACGGGGACCCTCCAATG |
| TEAD4-R | GCGAGCATACTCTGTCTCAAC |
| HIF1A-F | GAACGTCGAAAAGAAAAGTCTCG |
| HIF1A-R | CCTTATCAAGATGCGAACTCACA |
| Menin-F | GGGATGAACACATCTACCCCT |
| Menin-R | GTCTTCCCGGCAGTAGTTGTA |
| ChIP primers | |
| TEAD4(a)-F | GTCCTTAGCAGAGCCTTG |
| TEAD4(a)-R | CAACGCAATACACAGAACA |
| TEAD4(b)-F | GGTGTCTCTGTCCTTAGCA |
| TEAD4(b)-R | ACTCGCACAACGCAATAC |
| TEAD4(c)-F | GGCAGATGTGTTACTCCTA |
| TEAD4(c)-R | GAGTGGCTGGCAGTATAG |
| TEAD4(d)-F | GGCAGATGTGTTACTCCTA |
| TEAD4(d)-R | CTGGCAGTATAGGGACCT |
| MLL1(a)-F | GTCCTTAGCAGAGCCTTG |
| MLL1(a)-R | CAACGCAATACACAGAACA |
| MLL1(b)-F | ATAATGCCCAACGCTCTC |
| MLL1(b)-R | CTTCTTCCTCCGGTCTCT |
| MLL1(c)-F | GCGTTCAATTCGGGCTAA |
| MLL1(c)-R | AGAGCAGCTTCCAGTATAAC |
| MLL1(d)-F | GGCAGATGTGTTACTCCTA |
| MLL1(d)-R | CTGGCAGTATAGGGACCT |
| MEIS1-F | CCTCCTTCTCTAATCTCCTTC |
| MEIS1-R | CCTAATCAGTTTCTCTCTTCTC |
| TP53-F | GAATGGGTACGTCTGAGAAT |
| TP53-R | CGTCTGGAACTGGAATGG |

Table S3. Mouse primer information.

| Name | Sequences (5′ to 3′) |
| --- | --- |
| syna-F | CTCCAGGAGGCTAACTCTTCC |
| syna-R | TCCGGGCTGAGTACATGATTC |
| synb-F | ATGACAGGCTTTTGGGTCCTC |
| synb-R | GTTGGTATCACGTAGGATGTGG |
| gcm1-F | GATACTGAGCTGGGACATTAACG |
| gcm1-R | CTGTCGTCCGAGCTGTAGATG |
| actb-F | GTGACGTTGACATCCGTAAAGA |
| actb-R | GCCGGACTCATCGTACTCC |
