# Supplementary material for: Epigenetic drug screening for trophoblast syncytialization reveals a novel role for MLL1 in regulating fetoplacental growth
Source: BMC Med. 2024 Feb 5;22:57. doi: 10.1186/s12916-024-03264-8 (PMC10845764; doi:10.1186/s12916-024-03264-8)

Additional File 3: Original blot images.

Fig. 2B

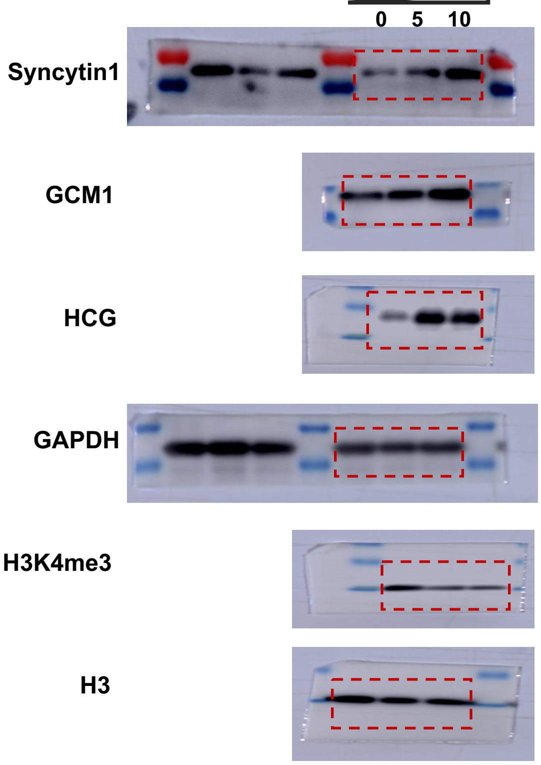

Fig. 2I

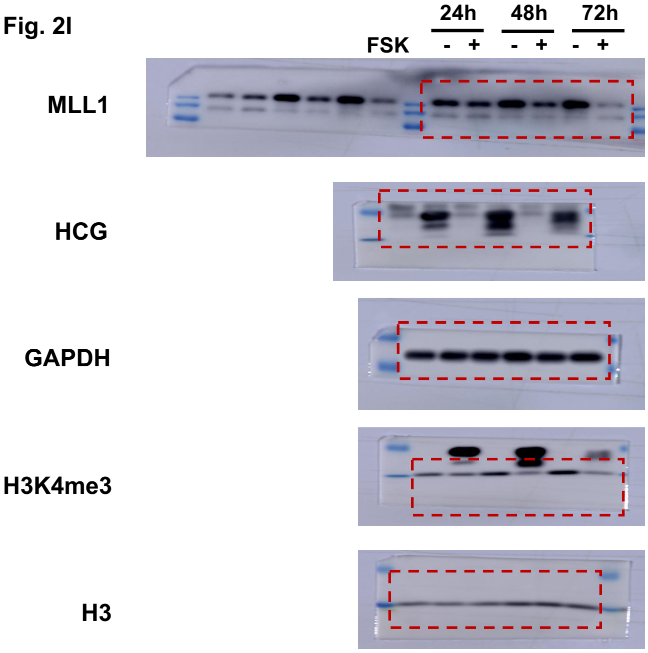

Fig. 2N

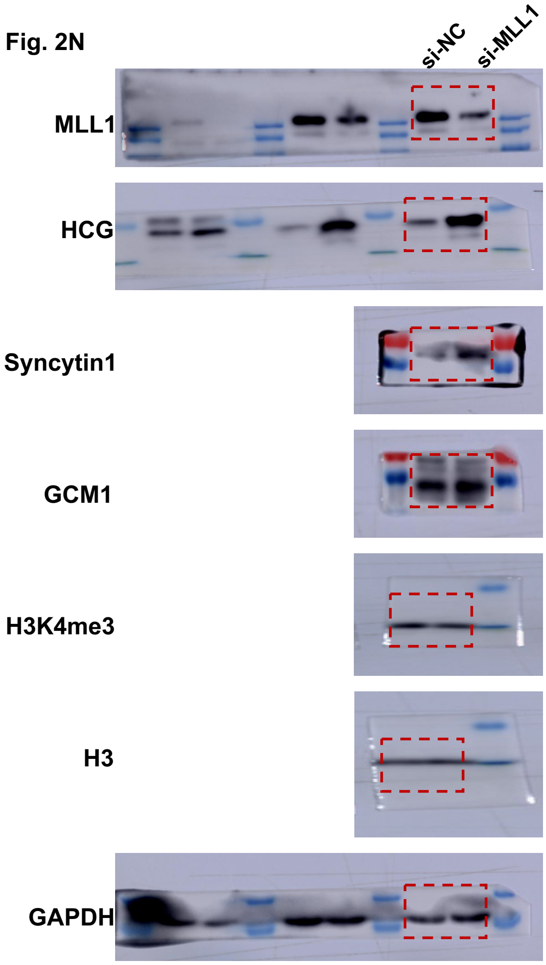

Fig. 3H

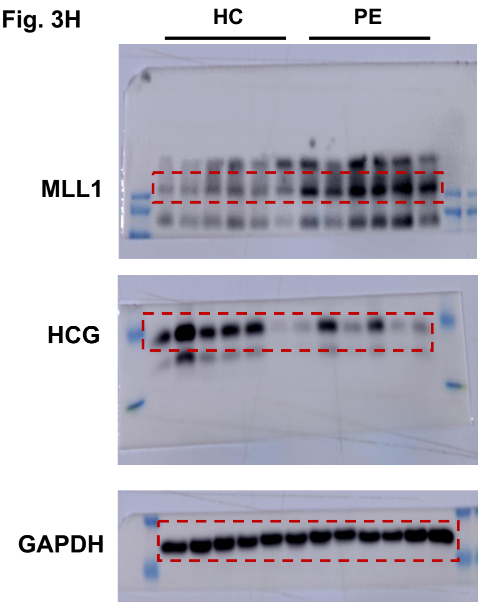

Fig. 5G

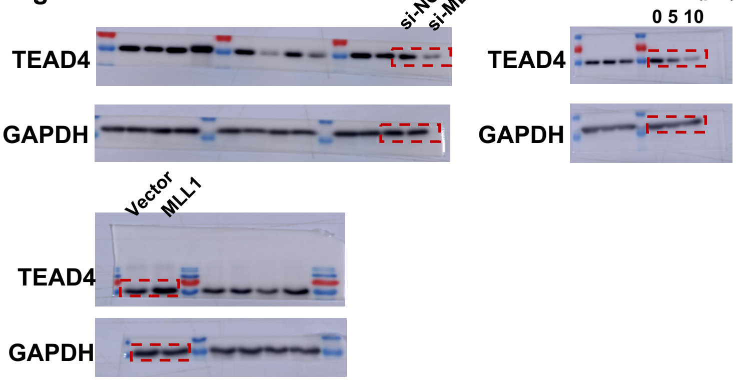

Fig. 5L

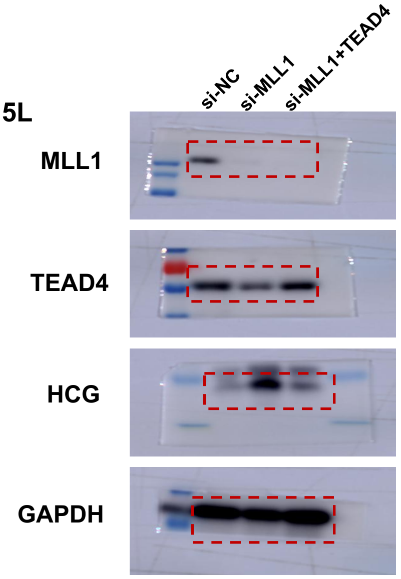

Fig. 6C

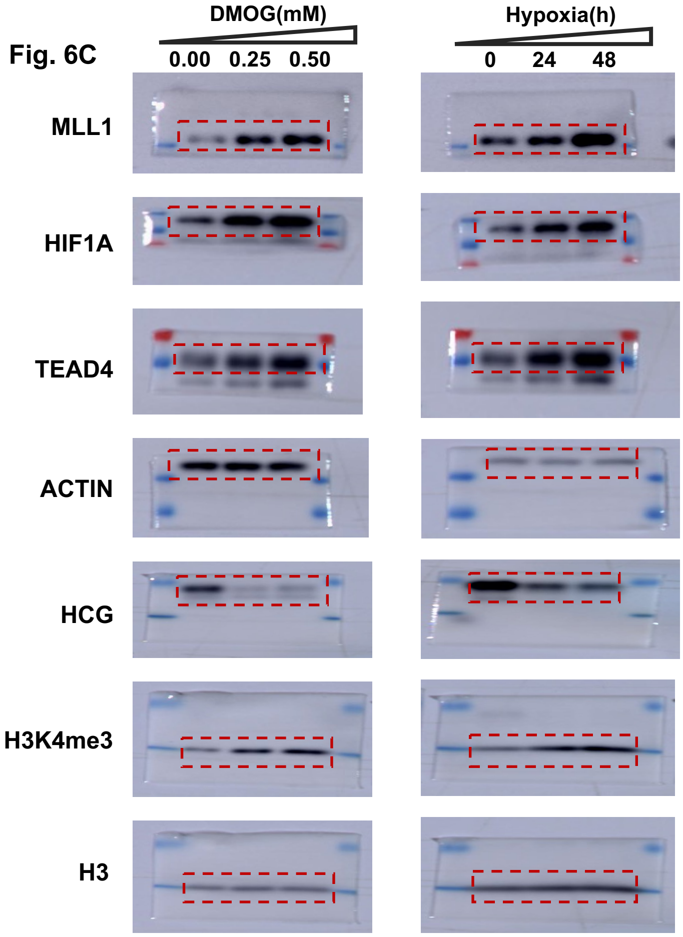

Fig. 6I

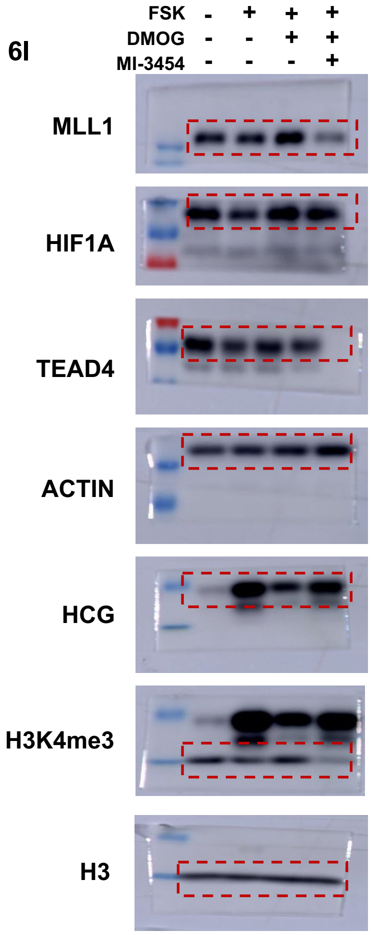

Fig. 7D

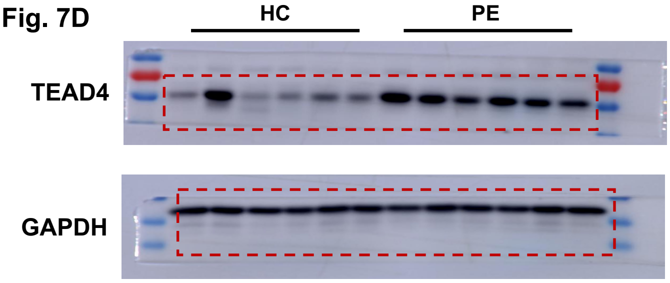

Fig. 7H

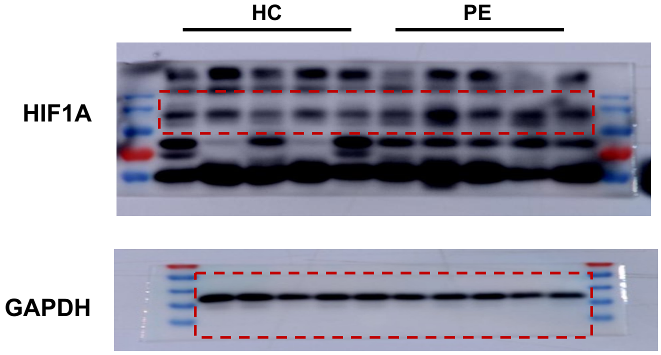

Fig. 8H

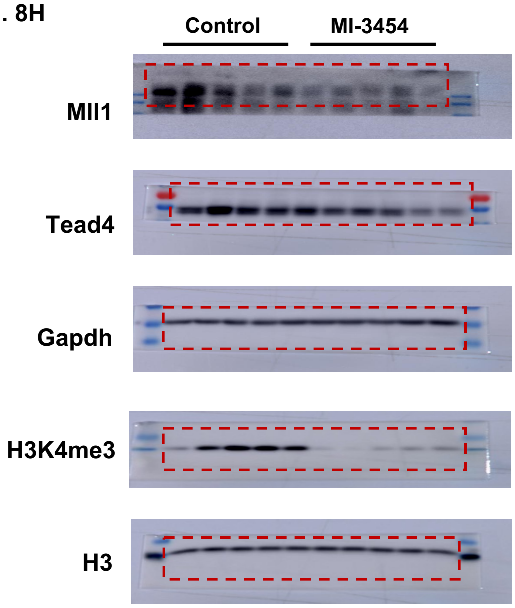

Fig. S2E

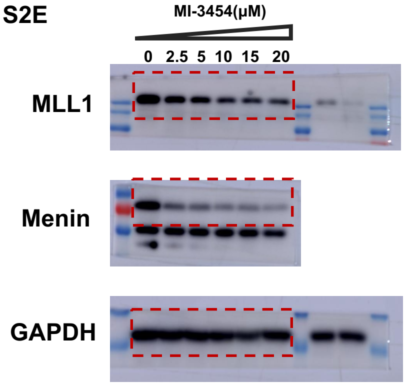

Fig. S3G

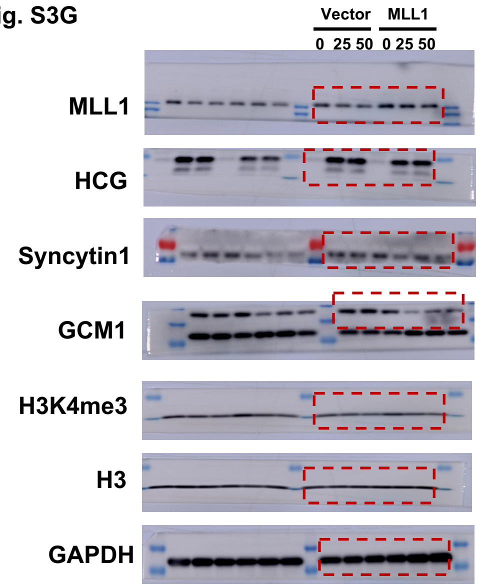

Fig. S3B

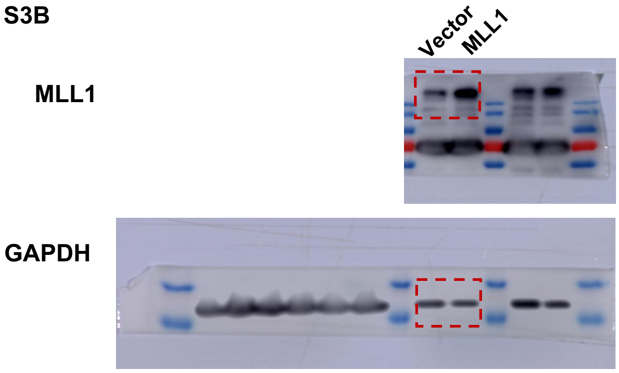

Fig. S6E

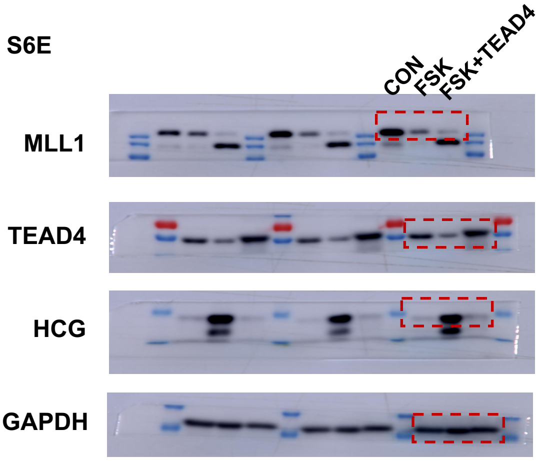

Supplement: Supplementary file 3 — Additional file 3. Original blot images. [file 12916_2024_3264_MOESM3_ESM.pdf]
